# Ultrasound‐Activated Nanoinhibitors Blocking the Chemo‐Immune Checkpoint to Overcome Chemotherapy‐Induced Immune Resistance

**DOI:** 10.1002/advs.77635

**Published:** 2026-09-08

**Authors:** Yunfan Liu, Chier Du, Leilei Zhu, Hongjin An, Shengzhe Hou, Yi Lan, Xuemei Tang, Min Xu, Peng Luo, Hua Teng, Hao Yang, Xun Guo, Jianli Ren, Zhiyi Zhou

**Affiliations:** ^1^ Department of Ultrasound The Second Affiliated Hospital of Chongqing Medical University Chongqing Key Laboratory of Ultrasound Molecular Imaging and Therapy Institute of Ultrasound Imaging Chongqing Medical University Chongqing China; ^2^ Department of Ultrasound The First Affiliated Hospital of Chongqing Medical University Chongqing China; ^3^ Center For Obesity Prevention and Treatment Chongqing Academy of Medical Sciences Chongqing General Hospital Chongqing University Chongqing China

**Keywords:** chemo‐immunotherapy, immune checkpoint blockade, siRNA and drug co‐delivery, ultrasound‐responsive nanomedicine

## Abstract

Breast cancer features an immunosuppressive tumor microenvironment following chemotherapy and develops resistance to conventional immune checkpoint inhibitors. Through single‐cell database mining and in vitro analysis, we demonstrate that chemotherapy upregulates Xkr8 expression and promotes phosphatidylserine (PS) externalization, which mediates macrophage‐ and dendritic cell (DC)‐intrinsic immunosuppression. To address this, we fabricate an ultrasound‐activated nanoinhibitor (Sono‐TT8) that co‐delivers tirapazamine (TPZ) and siXkr8 using a TiO_2_‐derived sonosensitizer functionalized with the tLyP‐1 peptide for active targeting of neuropilin‐1 (NRP‐1)‐overexpressing tumor cells. Upon ultrasound irradiation, Sono‐TT8 induces mitochondrial dysfunction and exacerbates local hypoxia to activate TPZ, while concurrently delivering siXkr8 to silence the Xkr8‐PS axis during chemotherapy‐induced apoptosis. This nanoinhibitor reprograms the tumor immunosuppressive microenvironment and reduces tumor growth. Furthermore, Sono‐TT8 exhibits superior tumor suppression and synergizes with αPD‐L1 to inhibit metastatic progression. Our work establishes a chemo‐immune checkpoint blockade strategy to overcome immune resistance in breast cancer, offering a nanosensitization approach compatible with multiple clinical treatment modalities.

## Graphical abstract

A sono‐responsive chemo‐immune checkpoint nanoinhibitor (Sono‐TT8) co‐delivering TPZ chemotherapy and siXkr8 simultaneously kills tumor cells and blocks PS externalization, reversing chemotherapy‐induced immunosuppression and enhancing antitumor immunity.

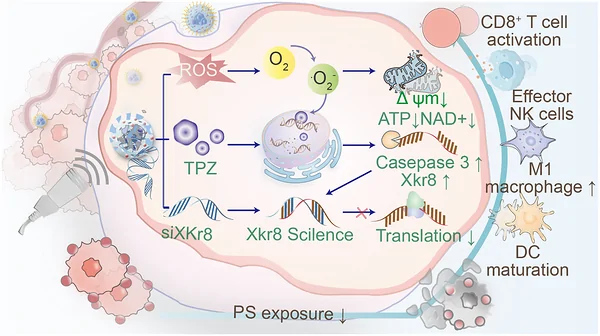

## 1 Introduction

Currently, chemotherapy remains the cornerstone of clinical treatment for breast cancer. However, its efficacy is frequently compromised by chemoresistance and systemic toxicity, which ultimately contribute to tumor recurrence and metastasis [1, 2]. Although immunotherapy has revolutionized the therapeutic landscape of breast cancer, the majority of patients derive limited benefit from immune checkpoint blockade (ICB) [3, 4, 5]. These observations suggest that chemotherapy may inadvertently counteract the antitumor immune response, thereby underscoring the urgent need for innovative strategies to overcome the intrinsic limitations of conventional chemotherapeutic regimens.

Considerable efforts have been devoted to elucidating the impact of chemotherapy on immune resistance in cancer cells [6, 7]. Mechanistically, chemotherapy induces apoptotic cell death primarily through DNA damage, culminating in caspase‐3 activation [8, 9]. As a critical scramblase, Xkr8 harbors a caspase‐3 recognition site in its C‐terminal region and is post‐transcriptionally activated by caspases during the apoptotic cascade [10, 11]. Upon activation, Xkr8 mediates the collapse of plasma membrane phospholipid asymmetry, randomizing phospholipid distribution across the bilayer and potently promoting phosphatidylserine (PS) externalization on the cell surface [10]. While surface‐exposed PS serves as a canonical “eat‐me” signal that facilitates efferocytosis of apoptotic debris [12, 13], it concurrently instigates a spectrum of immunosuppressive programs in the tumor microenvironment (TME) [14, 15]. Specifically, PS is recognized by cognate receptors on infiltrating immune cells, driving tumor‐associated macrophages toward an M2‐like immunosuppressive phenotype [10, 16], impairing dendritic cell antigen presentation [17], and restraining T cell activation [18, 19]. Therefore, these findings position the Xkr8‐PS as a chemo‐immune checkpoint that contributes to tumor immunosuppression by actively dampening antitumor immune responses, which may exert a more pronounced impact on therapeutic resistance than conventional immune checkpoints (e.g., PD‐1/PD‐L1) in the context of chemotherapeutic intervention. Although strategies aimed at blocking PS externalization, including annexin A5 or PS‐specific antibodies, have been explored to potentiate immune effects in chemotherapeutically treated patients [9, 18, 20], a phase III clinical trial failed to demonstrate superior outcomes over chemotherapy alone [21]. Moreover, to date, targeted approaches capable of directly silencing Xkr8 to bolster chemotherapy‐elicited antitumor immunity remain limited.

Nucleic acid‐based therapeutics, particularly messenger RNA (mRNA) and small interfering RNA (siRNA), have emerged as powerful modalities for disease intervention by virtue of their capacity to precisely modulate gene expression [22, 23, 24]. However, the in vivo application of siRNA remains encumbered by inherent limitations, including poor stability and insufficient cellular uptake. Recently, nanoparticle‐based siRNA delivery systems have shown considerable promise, offering high loading capacity, favorable biocompatibility, and the potential for functional surface engineering [25, 26, 27]. Importantly, such nanoplatforms can be rationally designed to integrate additional antitumor therapeutic modalities, thereby circumventing the constraints of monotherapy. Among these combinational strategies, sonodynamic therapy (SDT) has attracted growing interest as a non‐invasive treatment approach, which utilizes ultrasound irradiation to activate sonosensitizers‐either inorganic or organic‐transitioning them to an unstable high‐energy state, with subsequent energy transfer to surrounding molecular oxygen to generate cytotoxic reactive oxygen species (ROS) [28, 29]. Recent studies have further advanced SDT through the development of multifunctional nanosensitizer platforms that integrate drug delivery, immune modulation, and synergistic therapeutic modalities [30, 31, 32]. This ROS burst not only directly damages tumor cells but also elicits immunogenic cell death, offering a complementary avenue to counteract chemoresistance [29, 33]. Besides, the SDT process is often accompanied by aggravated tumor hypoxia, a feature that has been exploited to design hypoxia‐activated prodrugs for spatiotemporally controlled chemotherapy, thereby significantly mitigating the systemic toxicity typically associated with conventional chemotherapeutics and enhancing the overall therapeutic efficiency of the combined regimen [34].

**SCHEME 1:**
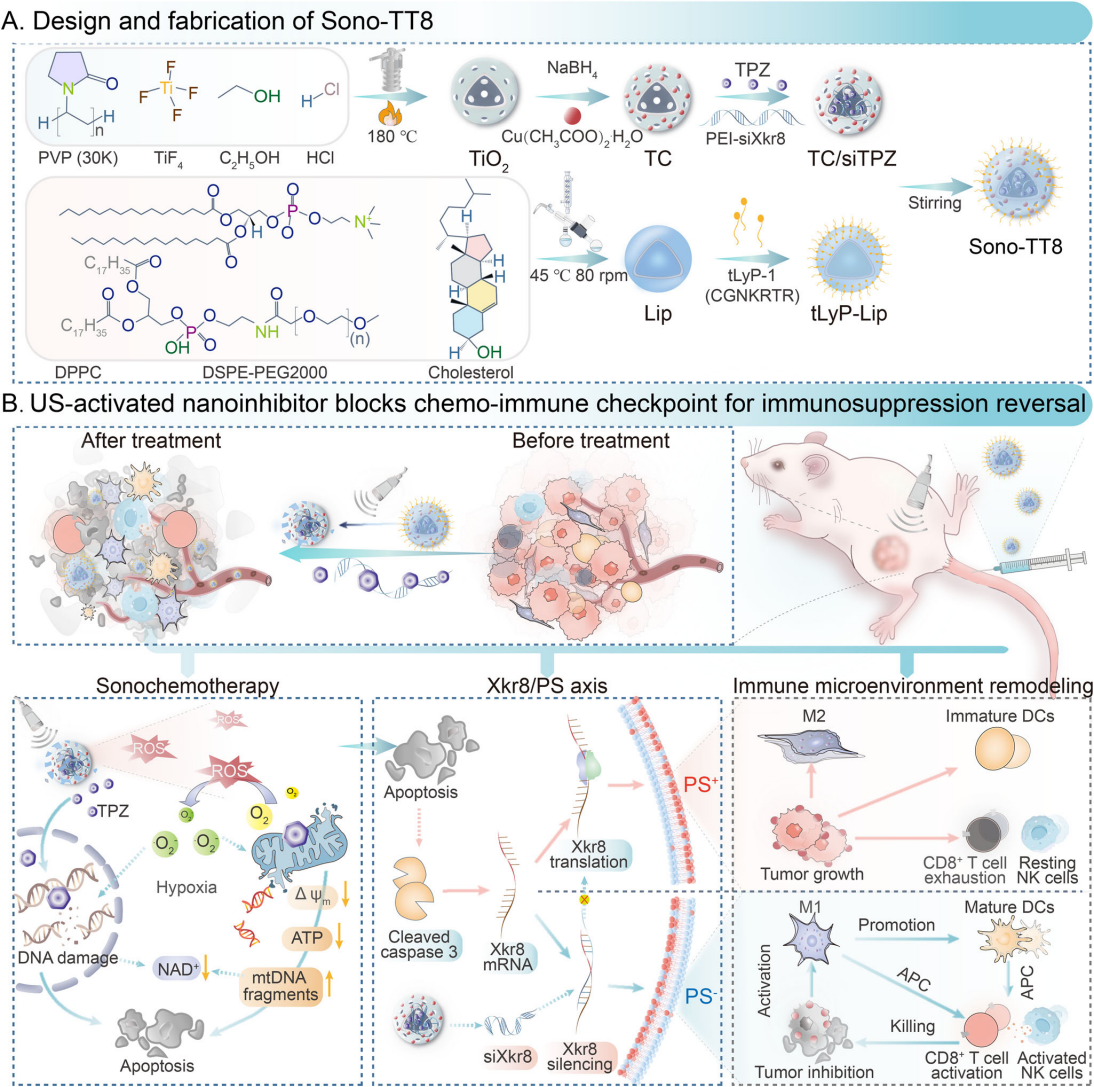
Schematic illustration of nanoinhibitor fabrication (A) and the proposed mechanism underlying chemo‐immune checkpoint blockade–mediated antitumor immunity (B).

Herein, we demonstrate that tirapazamine (TPZ), a hypoxia‐activated chemotherapeutic prodrug, upregulates Xkr8 expression and promotes PS externalization in breast cancer, which suppresses macrophage‐ and dendritic cell‐mediated immune signaling and drives immune resistance within the TME. To address the systemic toxicity and immunosuppressive effects of chemotherapy, we engineered a chemo‐immune checkpoint nanoinhibitor (CICN), designated Sono‐TT8, by co‐encapsulating TPZ and siXkr8 within a TiO_2_‐derived sonosensitizer platform and coating it with a tLyP‐1 (CGNKRTR)‐functionalized lipid membrane. The tLyP‐1 modification enables active targeting of neuropilin‐1 (NRP‐1)‐overexpressing tumor cells, while Sono‐TT8 integrates SDT‐unlocked chemotherapy and tumor‐cell‐specific Xkr8 downregulation to orchestrate the immune response. Benefiting from heterojunction doping, copper was deposited in situ onto the TiO_2_ surface to facilitate electron‐hole pair separation, substantially augmenting SDT‐mediated ROS generation and oxygen consumption. Upon cellular internalization, Sono‐TT8 responds to exogenous ultrasound irradiation to trigger TPZ activation and siXkr8 release, which concurrently induces tumor cell apoptosis and silences Xkr8 expression, thereby precluding caspase‐3‐mediated Xkr8 cleavage and subsequent PS externalization. This design effectively preserves direct tumoricidal activity while reversing Xkr8‐PS chemo‐immune checkpoint‐associated immunosuppression, as evidenced by macrophage repolarization toward the M1 phenotype, increased cDC1 abundance and antigen cross‐presentation, and enhanced T‐cell activation. Leveraging the sophisticated functionality of CICN, its combination with ultrasound (US) irradiation suppressed primary tumor growth, prevented lung metastasis, and enhanced the response to anti‐PD‐L1 (αPD‐L1) therapy. Collectively, our work proposes a synergistic therapeutic paradigm of chemo‐immune checkpoint blockade to potentiate breast cancer chemotherapy intervention (Scheme 1).

## 2 Results

### 2.1 Chemotherapy‐Induced Xkr8 Mediates PS Externalization to Drive Immunosuppression

**FIGURE 1:**
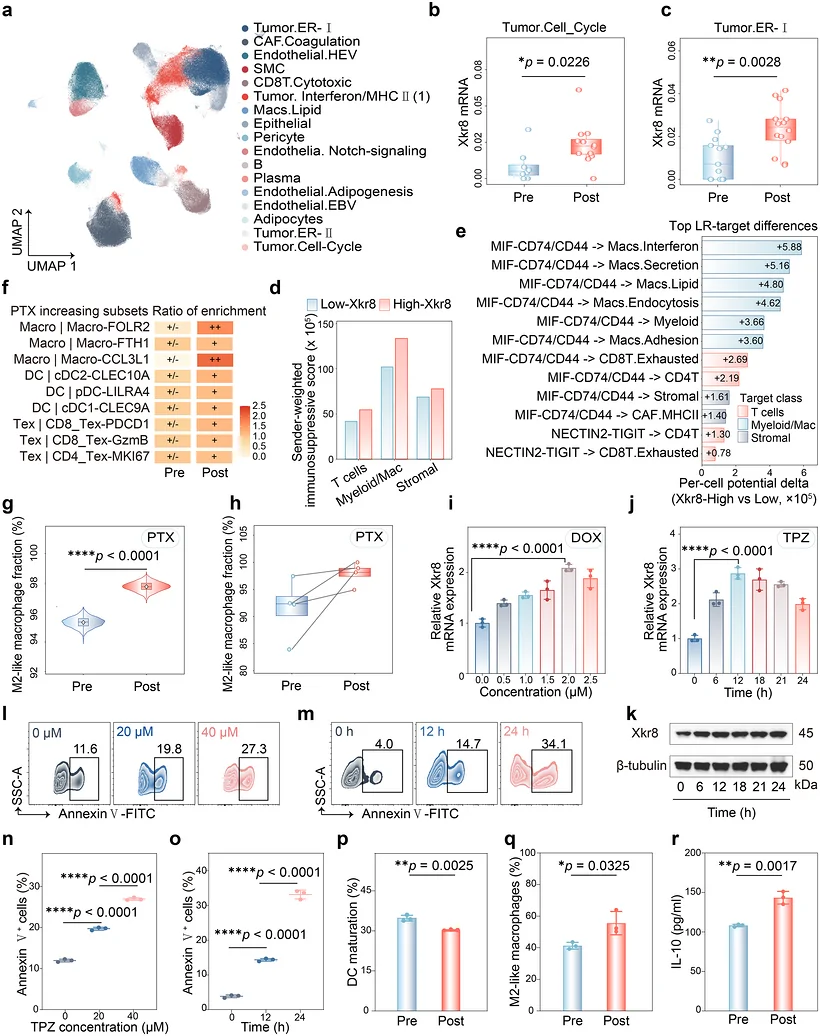
Chemotherapy‐induced Xkr8 upregulation and PS externalization drive immunosuppression. (a) UMAP plot of 17 tumor cell subsets identified by unsupervised clustering. (b,c) Xkr8 expression in ER‑I and cell‑cycle subsets before (Pre) and after (Post) Adriamycin/Cyclophosphamide (AC) therapy. (d) Per‑cell immunosuppressive scores of Xkr8‑High versus Xkr8‑Low tumor cells toward T cells, myeloid/macrophages, and stromal cells. (e) Per‑cell potential delta of immunosuppressive ligand‐receptor(L‐R) links between Xkr8‑High and Xkr8‑Low tumor cells. Positive values indicate enhanced signaling in the Xkr8‑High population. (f) Changes in immunosuppressive cell populations (macrophages, DCs, and T cells) in an independent paclitaxel (PTX)‑treated dataset (GSE169246). (g–h) M2‑like macrophage proportions before and after chemotherapy in paired and pooled analyses (*n*=4). (i,j) qRT‑PCR analysis of Xkr8 mRNA expression in 4T1 cells treated with (i) DOX (dose‑dependent) and (j) TPZ (time‑dependent) (*n*=3). (k) Western blot analysis of Xkr8 protein expression in 4T1 cells after TPZ treatment, with β‑tubulin as a loading control. (l–o) Flow cytometric analysis of PS externalization (Annexin V^+^) in 4T1 cells with increasing TPZ concentration or treatment time (*n*=3). (p) Quantitative analysis of DC maturation (CD80^+^ CD86^+^) after co‑culture of DC2.4 cells with TPZ‑pretreated or untreated 4T1 cells (*n*=3). (q) Quantitative analysis of M2‑like macrophage polarization (CD206^+^) after co‑culture of RAW264.7 macrophages with TPZ‑pretreated or untreated 4T1 cells (*n*=3). (r) ELISA measurement of IL‑10 secretion in macrophage supernatants after co‑culture with TPZ‑pretreated or untreated 4T1 cells (*n*=3). Statistical significance between two groups was determined using a two‑tailed unpaired Student's *t*‑test. (ns, not significant; *p < 0.05; **p < 0.01; ***p < 0.001; ****p < 0.0001).

Given that activation of the caspase‐3‐Xkr8‐PS axis is a hallmark of apoptotic cells, and chemotherapy induces extensive DNA damage‐triggered apoptosis, we hypothesized that chemotherapy may promote Xkr8 activation and thereby remodel the tumor immune microenvironment. To test this possibility, we analyzed a public single‐cell RNA sequencing dataset from breast cancer patients treated with Adriamycin/Cyclophosphamide (AC) (Zenodo: 10.5281/zenodo.15799565) [35]. Unsupervised clustering identified 17 tumor cell subsets (Figure 1a). Xkr8 expression was significantly upregulated after chemotherapy in the ER‑I (low estrogen receptor signaling) and cell‑cycle (proliferating) subsets (Figure 1b,c), consistent with the increased susceptibility of these populations to chemotherapy‐induced apoptosis. Notably, normalized per‑cell analysis revealed that Xkr8‑high tumor cells exhibited higher immunosuppressive scores toward T cells, myeloid/macrophages, and stromal cells compared with Xkr8‑low tumor cells (Figures 1d and S1). Consistently, CellChat analysis demonstrated enriched immunosuppressive ligand‑receptor signaling from Xkr8‑high tumor cells to macrophages and T cells (Figure 1e), suggesting that chemotherapy‐induced Xkr8 upregulation is associated with extensive immunosuppressive intercellular communication within the TME. To further validate these observations, we analyzed an independent paclitaxel (PTX)‑treated dataset (GSE169246). Chemotherapy consistently increased immunosuppressive subpopulations of macrophages, dendritic cells (DCs), and T cells, with macrophages showing the most prominent increase (Figure 1f). Both paired and pooled analyses confirmed a significant increase in M2‑like macrophages following chemotherapy (Figure 1g,h).

To complement the single‐cell findings, 4T1 cells were treated with doxorubicin (DOX) or TPZ. As shown in Figure 1i,j, DOX induced a dose‐dependent increase in Xkr8 mRNA expression, while TPZ elevated Xkr8 mRNA in a time‐dependent manner. Consistently, western blot analysis confirmed progressive upregulation of Xkr8 protein following TPZ treatment (Figure 1k). Flow cytometry further revealed that chemotherapy markedly increased cell‐surface PS exposure in both a dose‐ and time‐dependent manner (Figure 1l–o), indicating activation of the Xkr8‐PS axis in response to chemotherapy‐induced apoptosis. To investigate the immunological consequences of chemotherapy‐treated tumor cells, TPZ‐pretreated 4T1 cells were co‐cultured with DCs or macrophages. Notably, TPZ‑pretreated tumor cells significantly impaired DC maturation while promoting macrophage polarization toward an immunosuppressive M2‐like phenotype, accompanied by increased IL‐10 secretion (Figure 1p–r). Collectively, these results confirm that Xkr8 upregulation and PS externalization are general responses to apoptotic stimuli across different chemotherapeutic agents, rather than being specific to a particular drug, suggesting a broad role for the Xkr8‐PS axis in chemotherapy‑associated immune modulation.

### 2.2 Design, Fabrication, and Characterization of Sono‐TT8

To achieve targeted blockade of this chemo‑immune checkpoint, we developed a nanoinhibitor co‑encapsulating the hypoxia‑activated prodrug (TPZ) and siXkr8. The nanoplatform was constructed on an inorganic sonosensitizer (TiO_2_), which was synthesized via a polyvinylpyrrolidone (PVP)‐assisted solvothermal method using titanium tetrafluoride (TiF_4_) as the precursor. To enhance the efficacy of SDT, copper was deposited in situ onto the TiO_2_ surface (termed as TC) via an impregnation‐reduction process with sodium borohydride (NaBH_4_) as the reducing agent, followed by functionalization with tLyP‐1 targeting peptide‐modified lipid membrane.

**FIGURE 2:**
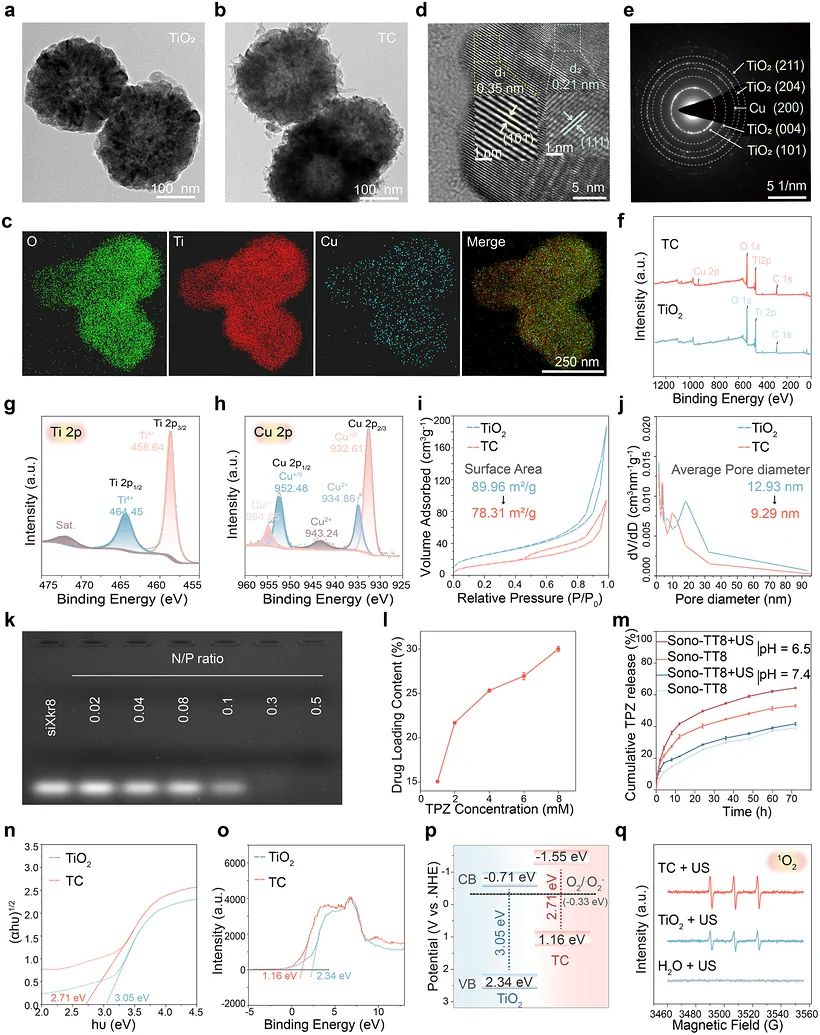
Preparation and characterization of Sono‐TT8. (a,b) TEM images of (a) TiO_2_ and (b) TC NPs. (Scale bars: 100 nm)_._ (c) Element mapping of TC NPs. (Scale bar: 250 nm). (d) High‐resolution TEM image of TC. The interplanar spacings of 0.35 and 0.21 nm represent the crystalline structure of TiO_2_ and Cu. (Scale bars: 5 nm; inset: 1 nm). (e) SAED pattern of TC. (f) XPS full survey spectrum of TiO_2_ and TC. (g) Ti 2p and (h) Cu 2p XPS spectrum of TC. (i) N_2_ adsorption‐desorption isotherms of TiO_2_ and TC. (j) Pore size distributions of TiO_2_ and TC. (k) Gel‐retardation assay of PEI‐siXkr8 complexes at various N/P ratios. (l) Drug loading content (DLC) of TPZ in Sono‐TT8. (m) Cumulative TPZ release from Sono‐TT8 NPs at pH 6.5 or 7.4, with or without US irradiation (*n*=3). (n) Band gap and (o) valence band of TiO_2_ and TC determined by UV‑Vis diffuse reflectance spectroscopy and VB‐XPS, respectively. (p) Schematic electronic band structures of TiO_2_ and TC. (q) ESR spectra of H_2_O, TiO_2_ and TC under US irradiation for detection of ^1^O_2_ generation. Results are presented as means ± SD.

Transmission electron microscopy (TEM) images revealed that both TiO_2_ and TC exhibited hollow mesoporous spherical morphologies with uniform sizes (Figure 2a,b). SEM imaging further confirmed the spherical morphology and uniform size distribution of TC nanoparticles (Figure S2a). The expected elements in TC, including titanium, oxygen, and copper, were all present in the elemental mapping images (Figure 2c). In addition, high‐resolution TEM (HRTEM) of TC showed distinct lattice fringes with interplanar spacings of 0.35 nm and 0.21 nm, corresponding to the [101]. plane of anatase TiO_2_ and the [111]. plane of face‐centered cubic Cu, respectively (Figure 2d). The selected‐area electron diffraction (SAED) pattern of TC displayed polycrystalline concentric rings (Figure 2e), matching the crystal lattices of both TiO_2_ and Cu. Furthermore, X‐ray diffraction (XRD) analysis of TC revealed diffraction patterns consistent with TiO_2_ (PDF#04‐002‐2751), while no distinct peaks attributable to metallic copper or copper oxides were observed (Figure S2b). X‐ray photoelectron spectroscopy (XPS) also confirmed the presence of carbon, oxygen, titanium, and copper in TC (Figure 2f). The Ti 2p spectrum exhibited two characteristic peaks at binding energies of 458.64 eV and 464.45 eV, corresponding to Ti 2p_3/2_ and Ti 2p_1/2_ of TiO_2_, respectively (Figure 2g). The Cu 2p spectrum displayed a dominant peak at 932.61 eV accompanied by weak satellite features (Figure 2h), indicating that copper exists predominantly in a reduced state (Cu^0^/Cu^+^), with only a minor fraction present as Cu^2+^. Nitrogen adsorption‐desorption isotherms indicated that both TiO_2_ and TC possessed mesoporous structures, with the Brunauer‐Emmett‐Teller specific surface area of TC (78.31 m^2^ g^−1^) being lower than that of TiO_2_ (89.96 m^2^ g^−1^) (Figure 2i). Simultaneously, the pore size of TC (9.29 nm) and TiO_2_ (12.93 nm) was detected (Figure 2j), which was favorable for subsequent loading of TPZ and siXkr8. However, naked siRNA is highly susceptible to nuclease degradation in physiological environments. To protect it during preparation and delivery, we pre‐complexed siRNA with polyethyleneimine (PEI) via electrostatic interactions, forming stable complexes before loading into the TC. The successful encapsulation was confirmed by gel electrophoresis (Figure 2k). We then calculated the loading content of TPZ and siXkr8, which reached 30.00 ± 0.31% and 5.98 ± 0.00%, respectively (Figures 2l and S2c), and elemental mapping revealed the presence of Ti, O, Cu, N, and P (Figure S2d). Subsequently, the nanoplatform was sealed with a tLyP‐1 peptide‐modified lipid membrane to enhance cargo delivery efficiency and prevent premature leakage. TEM showed that Sono‐TT8 remained spherical with a visible membrane coating (Figure S2e). Dynamic light scattering (DLS) measurements (Figure S2f,g) revealed hydrodynamic sizes of 233.13 ± 66.84 nm (TiO_2_), 246.47 ± 40.56 nm (TC), and 278.13 ± 50.55 nm (Sono‐TT8). Meanwhile, zeta potentials changed stepwise from 17.55 ± 0.21 mV (TiO_2_) to 5.55 ± 0.56 mV (TC) and then to 11.95 ± 1.21 mV (Sono‐TT8), confirming the sequential drug loading and surface modifications. Furthermore, Sono‑TT8 exhibited good colloidal stability, with particle size remaining around 300 nm over 9 days in PBS, water, and serum‑containing medium (Figure S2h). Finally, we systematically evaluated the spatiotemporal drug release profile. A buffer solution at pH 6.5 was selected to mimic the TME. Under neutral pH conditions, approximately 41.78 ± 0.72% of TPZ was released over 72 h. In contrast, at pH 6.5, the release efficiency reached 53.11± 0.57%, and upon combined ultrasound stimulation, the drug release further increased to 64.40 ± 0.31% (Figure 2m).

TPZ is a classic hypoxia‐activated chemotherapeutic agent, a feature that inherently minimizes the off‐target toxicity typically associated with conventional chemotherapy. To fully harness the spatiotemporal advantages of prodrug activation, we employed SDT to exacerbate oxygen depletion within the TME while simultaneously generating ROS for synergistic cancer treatment. Unfortunately, the sonodynamic efficiency of TiO_2_ is often limited by rapid electron‐hole recombination. We therefore systematically investigated the sonodynamic performance of the resulting TC composite. The band gaps of TiO_2_ and TC were determined to be 3.05 eV and 2.71 eV, respectively (Figure 2n). Compared with pristine TiO_2_, the narrower band gap of TC is likely attributable to the incorporation of Cu^+^ ions into the TiO_2_ lattice, facilitating electron‐hole separation and consequently enhancing ultrasound‐triggered ROS generation. Valence band XPS (VB‐XPS) revealed that the valence band edge of TC (1.16 eV vs. NHE) is significantly upshifted compared to that of pristine TiO_2_ (2.34 eV vs. NHE) (Figure 2o), and the conduction band edges were calculated to be −0.71 eV for TiO_2_ and −1.55 eV for TC (Figure 2p). Accordingly, electron spin resonance (ESR) spectroscopy was performed using TEMP as a spin trap. Under ultrasound irradiation, TC exhibited a characteristic 1:1:1 triplet signal corresponding to the TEMP‐^1^O_2_ adduct, whereas TiO_2_ showed a much weaker signal (Figure 2q). Altogether, these results demonstrate that TC promotes ultrasound‐triggered ROS production, which accelerates local oxygen consumption and thus creates a hypoxic environment conducive to TPZ activation.

### 2.3 Antitumor Performance and Mechanism of Sono‐TT8 In Vitro

**FIGURE 3:**
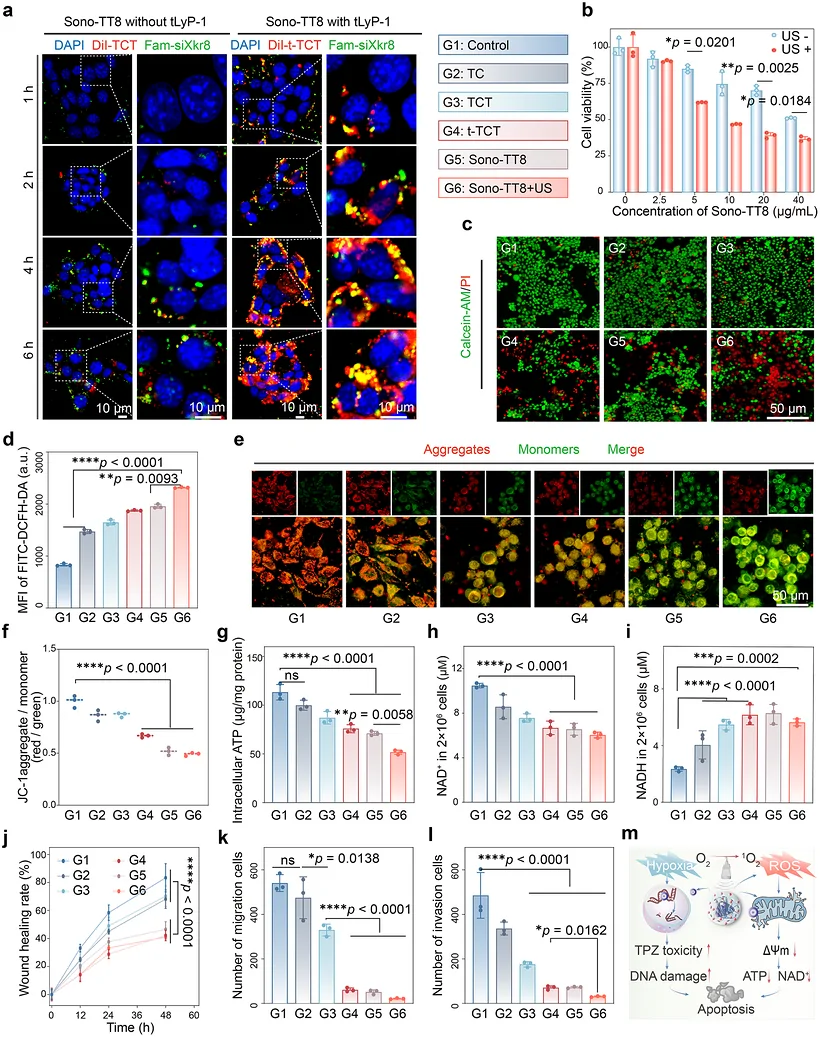
Therapeutic efficacy and mechanistic characterization of Sono‐TT8. (a) CLSM images of 4T1 cells incubated with targeted Sono‐TT8 or non‑targeted group for 1, 2, 4, and 6 h (scale bars: 10 µm). (b) CCK‐8 assay showing viability of 4T1 cells after treatment with Sono‐TT8 with or without US irradiation (*n*=3). (c) Live/dead fluorescence staining of 4T1 cells after various treatments; green and red fluorescence indicate live and dead cells, respectively (scale bar: 50 µm). (d) Flow cytometric analysis of intracellular ROS levels using DCFH‐DA probe (*n*=3). (e) CLSM images of JC‐1‐stained 4T1 cells after various treatments (scale bar: 50 µm). (f) Quantitative analysis of mitochondrial membrane potential (red/green fluorescence ratio) by flow cytometry (*n*=3). (g–i) Intracellular ATP (g), NAD^+^ (h), and NADH (i) levels in 4T1 cells after various treatments (*n*=3). (j–l) Scratch wound‐healing (j), Transwell migration (k), and invasion assays (l) (*n*=3). (m) Schematic illustration of the mechanical cytotoxicity of Sono‐TT8. Results are presented as means ± SD. Statistical significance was calculated by one‐way ANOVA with Tukey's multiple comparisons test. (ns, not significant; *p < 0.05; **p < 0.01; ***p < 0.001; ****p < 0.0001).

tLyP‐1 is a tumor‐homing and tissue‐penetrating peptide that exerts its targeting capability primarily through binding to NRP‐1 and activation of the canonical CendR (C‐end Rule) pathway [36]. To verify the contribution of tLyP‐1 to cellular internalization, we investigated the uptake behavior of Sono‐TT8 in 4T1 cells. Confocal laser scanning microscopy (CLSM) revealed a time‐dependent increase in intracellular accumulation of Sono‐TT8. The nanoinhibitors initially localized on the cell membrane and were subsequently internalized into the cytoplasm. Notably, the green fluorescence emitted by FAM‐labeled siXkr8 exhibited substantial colocalization with the red fluorescence of Dil‐labeled Sono‐TT8, demonstrating the successful encapsulation and intracellular delivery of siXkr8 by the nanoinhibitor. By contrast, 4T1 cells treated with the non‐tLyP‐1‐modified counterpart displayed markedly weaker red and green fluorescence signals after 6 h of incubation (Figure 3a). Consistent with the CLSM observations, flow cytometric analysis further confirmed the enhanced internalization efficiency of Sono‐TT8 in 4T1 cells (Figure S3a–c). Next, we evaluated the cytotoxicity of the nanoinhibitor toward tumor cells. The following groups were included: G1, Control; G2, TC; G3, TCT (TC loaded with TPZ); G4, t‑TCT (TC loaded with TPZ and modified with tLyP‑1); G5, Sono‐TT8; and G6, Sono‐TT8+US. To rule out the potential contribution of cuproptosis to the therapeutic outcome, we first examined FDX1 expression by Western blot. As shown in Figure S3d,e, FDX1 levels in the TC and Sono‑TT8 groups were comparable to those in the control group, suggesting that the Cu component does not induce detectable cuproptosis. Consistently, the TC exhibited negligible cytotoxicity toward both HUVECs and 4T1 cells at concentrations up to 40 µg mL^−1^ (Figure S3f). Contrastingly, loading of siXkr8 and TPZ (G5) significantly reduced the viability of 4T1 cells to 51.04 ± 0.37%. Upon further ultrasound irradiation (G6), cell viability decreased to approximately 36.61 ± 1.66% (Figure 3b). Moreover, the antitumor efficacy of the different formulations was visualized by Live/Dead staining. From the fluorescence images (Figure 3c), TC and TCT groups induced minimal cell death. In comparison, both t‐TCT and Sono‐TT8 groups elicited moderate tumor cell killing. Notably, ultrasound‐activated Sono‐TT8 produced the most pronounced cytotoxic effect, as evidenced by the substantial increase in dead‐cell staining. This improved therapeutic efficacy is likely attributable to the synergistic action of SDT‐triggered ROS and the efficient activation of TPZ [34, 37].

To elucidate the underlying therapeutic mechanism, intracellular ROS generation was evaluated using the DCFH‐DA probe. Flow cytometric analysis revealed that the Sono‐TT8+US group exhibited the highest green fluorescence intensity. In comparison, only a slight increase in ROS levels was observed in the other treatment groups (Figures 3d and S3g). Furthermore, mitochondrial superoxide production was evaluated using MitoSOX‐Red staining. Flow cytometric analysis revealed that Sono‐TT8+US treatment induced the most pronounced increase in MitoSOX fluorescence intensity among all groups (Figure S3h,i), demonstrating that this strategy effectively enhances mitochondrial oxidative stress. Given the pivotal role of mitochondria in ROS‐mediated cell death, we investigated mitochondrial function following treatment. JC‐1 staining demonstrated a pronounced increase in green fluorescence accompanied by a marked reduction in red fluorescence in cells treated with Sono‐TT8+US, indicative of substantial mitochondrial membrane depolarization (Figure 3e). Consistently, flow cytometric quantification showed that the Sono‐TT8+US group displayed the lowest red‐to‐green fluorescence ratio (aggregate/monomer), reaching 0.490 ± 0.016 (Figures 3f and S3j). Concomitantly, intracellular ATP levels were significantly reduced in the Sono‐TT8+US group compared with all other treatment groups (Figure 3g). Furthermore, this group exhibited a marked depletion of NAD^+^ accompanied by an accumulation of NADH (Figure 3h,i), collectively indicating impaired mitochondrial respiration and disruption of cellular energy homeostasis. Subsequently, we assessed the effects of Sono‐TT8 on tumor cell motility. Scratch wound‐healing and Transwell migration/invasion assays demonstrated that Sono‐TT8+US effectively inhibited wound closure, cell migration, and invasive behavior among all groups (Figures 3j–l and S3k–m), suggesting a substantial attenuation of the metastatic potential of 4T1 cells. Based on these findings, a mechanistic model for the ultrasound‐enhanced nanoinhibitor is proposed (Figure 3m), which elicits a dual‐hit therapeutic effect by simultaneously disrupting mitochondrial bioenergetics and amplifying TPZ‐mediated genotoxic stress.

### 2.4 Xkr8 Silencing Relieves PS Externalization‐Mediated Immunosuppression

**FIGURE 4:**
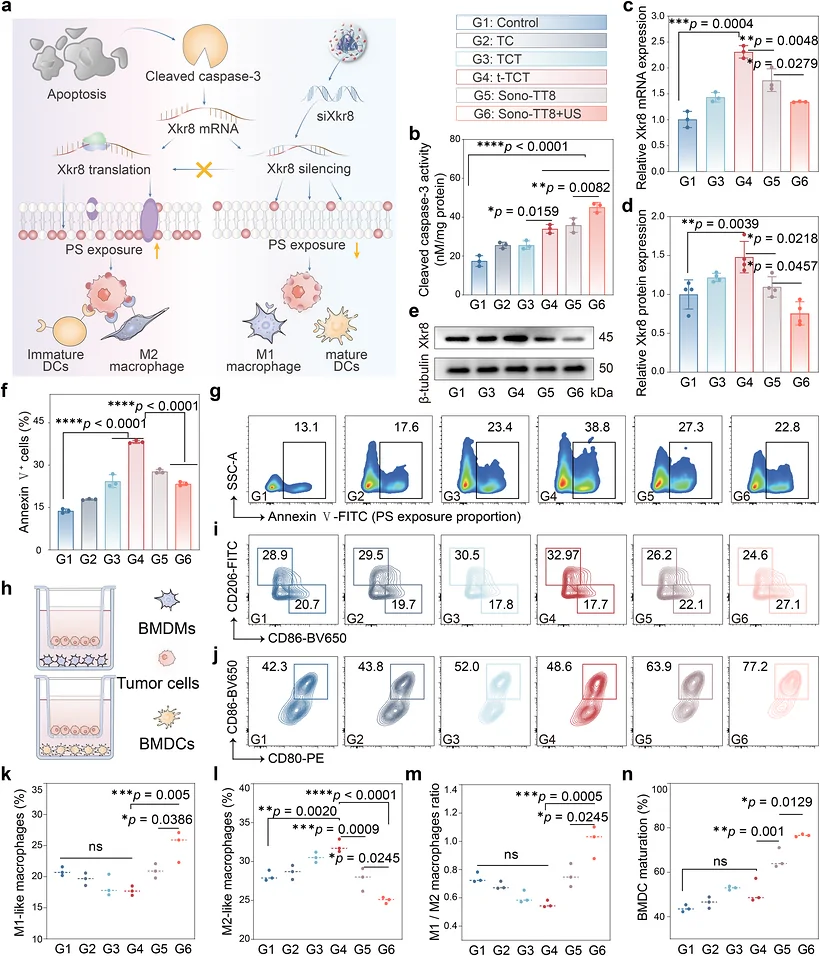
Xkr8 silencing relieves PS‑mediated immunosuppression to restore antitumor immunity. (a) Schematic diagram of Sono‐TT8‑mediated chemo‑immune checkpoint blockade for reversing immunosuppression. (b) Cleaved caspase‑3 activity in 4T1 cells after various treatments determined by colorimetric assay (*n*=3). (c) qRT‑PCR analysis of Xkr8 mRNA expression in 4T1 cells treated with PBS, TCT, t‑TCT, Sono‐TT8, and Sono‐TT8+US (*n*=3). (d,e) Western blot analysis of Xkr8 protein expression in 4T1 cells after different treatments, with β‐tubulin as a loading control (*n*=3). (f,g) Flow cytometric analysis of PS externalization (Annexin V^+^) in 4T1 cells in vitro (*n*=3). (h) Schematic representation of the co‑culture system involving 4T1 cells, BMDMs, and BMDCs. (i,j) Representative flow cytometric analysis of BMDM polarization (i) and BMDC maturation (j) in different groups. (k–n) Corresponding quantitative analysis of M1‑like macrophages (CD11b^+^ F4/80^+^ CD86^+^ CD206^−^) (k), M2‑like macrophages (CD11b^+^ F4/80^+^ CD86^−^ CD206^+^) (l), M1/M2 macrophage ratio (m), and DC maturation (CD11c^+^ CD80^+^ CD86^+^) (n) across different groups (*n*=3). Results are presented as means ± SD. Statistical significance was calculated by one‐way ANOVA with Tukey's multiple comparisons test. (ns, not significant; *p < 0.05; **p < 0.01; ***p < 0.001; ****p < 0.0001).

Our previous single‐cell transcriptomic analyses and in vitro studies demonstrated that chemotherapy induces the activation of caspase‐3, which subsequently cleaves Xkr8 and drives irreversible PS externalization during apoptosis. In parallel, exposed PS actively suppresses antitumor immunity and promotes the establishment of an immunosuppressive TME. Based on this rationale, we investigated whether the US‐activated nanoinhibitor could block the chemo‐immune checkpoint for enhancing immune activation (Figure 4a). We first evaluated the activation of caspase‐3 in 4T1 cells following various treatments. Colorimetric analysis showed that cleaved caspase‐3 levels were significantly elevated in all TPZ‐containing groups, including t‐TCT, Sono‐TT8, and Sono‐TT8+US, compared with the control and TC groups (Figure 4b). Among them, the Sono‐TT8+US group exhibited the highest level of caspase‐3 activation, whereas no significant difference was observed between the t‐TCT and Sono‐TT8 groups. These findings were consistent with the cytotoxicity results described above (Figure 3b,c). Subsequently, Xkr8 expression and PS externalization were examined. As shown in Figure 4c, qPCR analysis revealed that t‐TCT treatment markedly upregulated Xkr8 transcription, with Xkr8 mRNA levels increasing by 2.3‐fold relative to the control group. Conversely, incorporation of siXkr8 effectively suppressed Xkr8 expression in the Sono‐TT8 and Sono‐TT8+US groups. Notably, the enhanced silencing effect observed in the Sono‐TT8+US group may be attributed to US‐facilitated intracellular release of siXkr8. In addition, western blot analysis further corroborated these findings. Consistent with the transcriptional results, the t‐TCT group exhibited the highest Xkr8 protein expression, whereas introduction of siXkr8 markedly attenuated Xkr8 levels in both Sono‐TT8‐treated groups (Figure 4d,e). To determine whether Xkr8 silencing could suppress PS externalization, we quantified membrane‐exposed PS using Annexin V staining. Annexin V specifically binds extracellularly exposed PS and is widely used as a marker of apoptotic membrane remodeling. Flow cytometric analysis revealed that the proportion of PS‐positive cells increased 2.77‐fold in the t‐TCT group compared with the control group. Importantly, delivery of siXkr8 significantly reduced PS exposure, with the percentage of PS‐positive cells decreasing to 27.77 ± 0.72% and 23.40 ± 0.66% in the Sono‐TT8 and Sono‐TT8+US groups, respectively (Figure 4f,g). These results demonstrate that siXkr8 effectively blocks TPZ‐induced PS externalization, thereby alleviating chemotherapy‐associated immunosuppressive signaling.

Surface‐exposed PS on apoptotic tumor cells has been widely recognized as a potent immunosuppressive signal. On one hand, PS serves as an engulfment cue for macrophages, promoting efferocytosis while simultaneously inducing the secretion of anti‐inflammatory cytokines such as Interleukin‐10 (IL‐10) and driving macrophage polarization toward an immunosuppressive M2 phenotype [10]. In addition, PS can engage T‐cell immunoglobulin and mucin‐domain containing (TIM) family receptors expressed on DCs, suppressing pro‐inflammatory signaling pathways including NF‐κB and PI3K/Akt, ultimately impairing DC maturation, co‐stimulatory molecule expression, and antigen‐presenting capacity [15, 17]. To investigate the immunological consequences of Xkr8 silencing, we established Transwell co‐culture systems consisting of pretreated 4T1 tumor cells and either bone marrow‐derived macrophages (BMDMs) or bone marrow‐derived dendritic cells (BMDCs) isolated from BALB/c mice (Figure 4h). Flow cytometric analysis revealed that BMDMs co‐cultured with tumor cells treated with TC, TCT, or t‐TCT predominantly exhibited an M2‐like phenotype, characterized by elevated CD206 expression. In contrast, incorporation of siXkr8 effectively reversed this immunosuppressive phenotype. Both Sono‐TT8 and Sono‐TT8+US treatments markedly reduced CD206 expression and promoted macrophage repolarization toward a pro‐inflammatory M1 phenotype. Consequently, the M1/M2 ratio in the Sono‐TT8+US group reached 1.004 ± 0.114, representing an approximately 1.81‑fold and 1.36‑fold increase compared with the t‐TCT and control groups, respectively (Figure 4i,k–m). Consistent with this phenotypic reprogramming, marked alterations in BMDMs morphology were observed under bright‐field microscopy (Figure S4). BMDMs co‐cultured with TC‐, TCT‐, or t‐TCT‐treated tumor cells displayed an elongated spindle‐like morphology characteristic of M2 macrophages. By comparison, BMDMs exposed to tumor cells treated with siXkr8‐containing formulations predominantly adopted a rounded morphology, a feature commonly associated with inflammatory M1 macrophages. We then examined the effects of chemo‐immune checkpoint blockade on DC maturation. As expected, BMDCs co‐cultured with tumor cells from the non‐siXkr8 treatment groups exhibited relatively low expression of the co‐stimulatory molecules CD80 and CD86. Remarkably, co‐culture with Sono‐TT8‐ and Sono‐TT8+US‐treated tumor cells significantly enhanced BMDC maturation, increasing the proportion of CD80^+^ CD86^+^ cells to 65.90 ± 4.54% and 76.60 ± 0.60%, respectively, corresponding to 1.51‐fold and 1.82‐fold increases over the control group (Figure 4j,n). Collectively, these findings demonstrate that Xkr8 silencing effectively suppresses chemotherapy‐induced PS externalization, thereby relieving chemo‐immune checkpoint‐dependent immunosuppressive signaling.

### 2.5 US‐Activated Nanoinhibitor Potentiates Chemotherapeutic Efficacy in Breast Cancer

**FIGURE 5:**
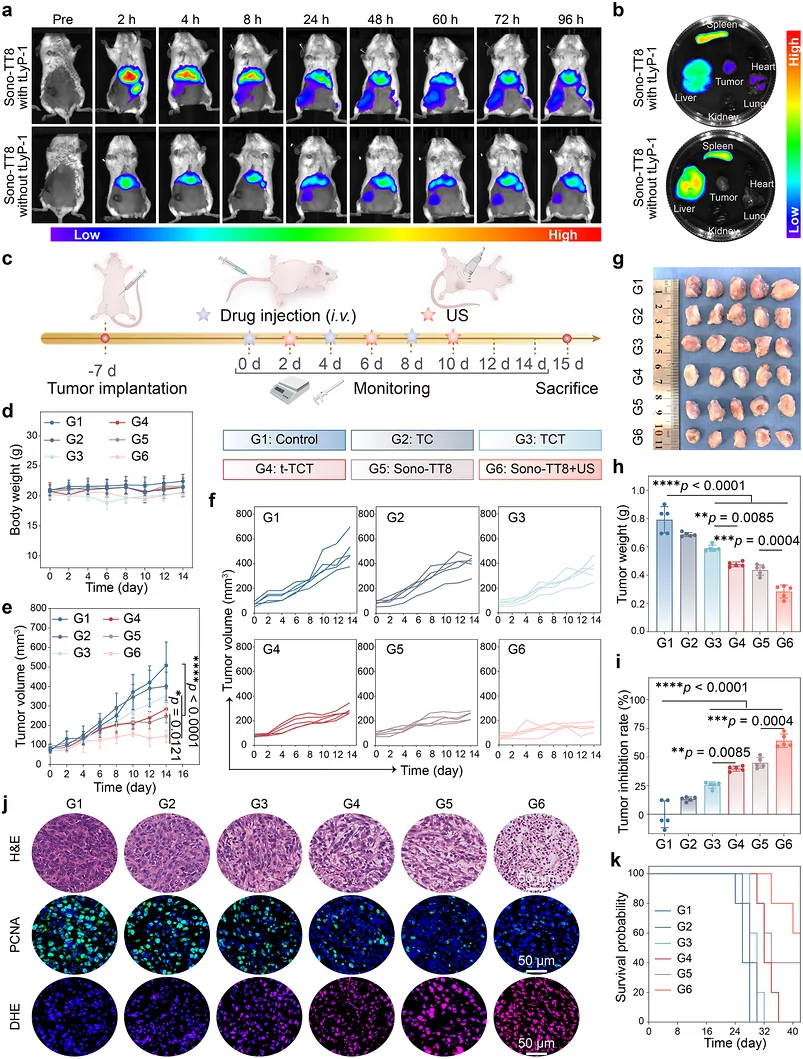
In vivo biodistribution and antitumor efficacy of Sono‐TT8 in 4T1 tumor‐bearing mice. (a) Real‐time fluorescence images of mice at various time points after intravenous administration of DiR‐labeled Sono‐TT8 (targeted) or its non‐targeted counterpart. (b) *Ex vivo* fluorescence imaging of major organs (heart, liver, spleen, lung, and kidney) and tumors at 96 h post‐injection. (c) Schematic illustration of the treatment schedule for 4T1 tumor‐bearing mice. (d,e) Body weight changes (d) and tumor volume growth curves (e) of mice after different treatments (*n*=5). (f) Individual tumor growth curves for each treatment group (*n*=5). (g) Representative photographs of dissected tumors from each group (*n*=5). (h,i) Tumor weights (h) and tumor inhibition rates (i) after various treatments (*n*=5). (j) H&E, PCNA, and DHE staining of tumor sections from different groups (scale bars: 50 µm). (k) Survival curves of mice in each treatment group (*n*=5). Results are presented as means ± SD. Statistical significance was calculated by one‐way ANOVA with Tukey's multiple comparisons test. (ns, not significant; *p < 0.05; **p < 0.01; ***p < 0.001; ****p < 0.0001).

Encouraged by the potent antitumor activity of Sono‐TT8 observed in vitro, we next investigated its biosafety profile prior to evaluating its therapeutic efficacy in vivo. BALB/c mice were intravenously administered Sono‐TT8 at a therapeutic dose of 20 mg kg^−1^, corresponding to an estimated peak blood concentration of approximately 267 µg mL^−1^. Blood samples and major organs were collected at predetermined time points (days 1, 3, 7, 14, and 28) following administration. Routine hematological and serum biochemical analyses revealed no significant abnormalities in Sono‐TT8‐treated mice compared with the control group throughout the observation period (Figure S5a,b). In addition, histological examination of major organs, including the heart, liver, spleen, lungs, and kidneys, showed no evident pathological abnormalities, tissue damage, or inflammatory infiltration following Sono‐TT8 treatment (Figure S5c). Then, we systematically investigated the in vivo biodistribution and tumor‐targeting capability of Sono‐TT8. The fluorescence intensity in blood samples showed a time‑dependent decrease (Figure S6a), with 33.76 ± 2.76% of the initial signal remaining at 48 h and 25.27 ± 4.20% at 72 h post‑injection, indicating that a substantial portion of the nanoparticles persisted in the circulation. This sustained circulation is favorable for improving tumor drug accumulation and extending the therapeutic window. As shown in Figures 5a and S6b, the fluorescence signal within tumors in the targeted group gradually increased over time, reaching a maximum intensity at 60 h post‐injection. Although the signal subsequently declined, substantial fluorescence remained detectable in the tumor region even at 96 h, indicating prolonged intratumoral retention. However, the non‐targeted group consistently exhibited markedly weaker tumor‐associated fluorescence throughout the observation period. Accordingly, 60 h post‐injection was selected as the optimal time point for subsequent ultrasound treatment. To further estimate the biodistribution profile, mice were sacrificed, and major organs together with tumors were harvested for *ex vivo* fluorescence imaging (Figures 5b and S6c). Both groups exhibited strong fluorescence signals in the liver and spleen. Notably, a readily visible fluorescence signal was observed in tumors from the Sono‐TT8 group, whereas minimal fluorescence was detected in tumors from the non‐targeted group. Quantitative analysis revealed that the tumor fluorescence intensity of Sono‐TT8 was approximately 3.73‐fold higher than that of the non‐targeted control.

Following the identification of Sono‐TT8 as an effective platform for enhancing chemotherapeutic efficacy and tumor‐targeted drug delivery, we next evaluated its therapeutic performance in vivo using a 4T1 tumor‐bearing mouse model (Figure 5c). Throughout the 14‐day treatment period, no appreciable body weight loss was observed in any treatment group, indicating the favorable tolerability of the administered formulations (Figure 5d). Tumor growth monitoring revealed that TC and TCT marginally inhibited tumor growth, whereas t‐TCT and Sono‐TT8 exhibited enhanced tumor growth inhibition. Importantly, US‐activated Sono‐TT8 achieved the most pronounced therapeutic outcome, effectively suppressing tumor progression (Figure 5e,f). Consistent with the tumor growth curves, representative photographs of excised tumors clearly showed that tumors from the Sono‐TT8+US group were substantially smaller than those from all other treatment groups (Figure 5g). Quantitative analysis further demonstrated that the average tumor weight in the Sono‐TT8+US group was significantly reduced (Figure 5h), corresponding to a tumor inhibition rate of 64.19 ± 5.79% (Figure 5i). To investigate the therapeutic response at the tissue level, hematoxylin and eosin (H&E), proliferating cell nuclear antigen (PCNA), and dihydroethidium (DHE) staining were performed on harvested tumor tissues (Figure 5j). Among all treatment groups, Sono‐TT8+US induced the most extensive histopathological damage, exhibited the lowest level of PCNA‐positive proliferating cells, and generated the strongest DHE fluorescence signal. Moreover, Kaplan‐Meier analysis demonstrated that mice receiving Sono‐TT8+US treatment exhibited markedly prolonged survival compared with all other groups (Figure 5k).

### 2.6 Blockade of Chemo‐Immune Checkpoint for Restoring Innate and Adaptive Antitumor Immunity

**FIGURE 6:**
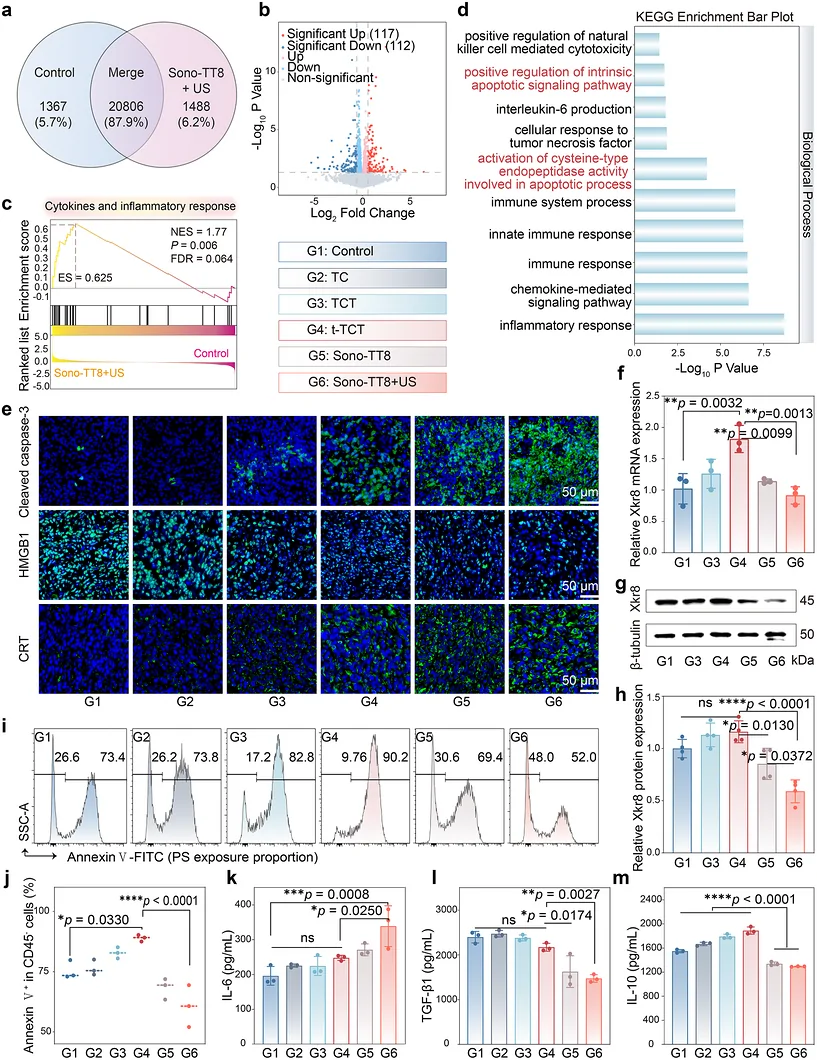
Mechanistic validation of chemo‐immune checkpoint blockade in reversing chemotherapy‑associated immunosuppression in vivo. (a) Venn diagram showing overlapping genes between the control and Sono‐TT8+US groups. (b) Volcano plot of differentially expressed genes in 4T1 tumors after treatment with PBS or Sono‐TT8+US (*n*=3). (c) GSEA of RNA‑seq data showing enrichment of the cytokines and inflammatory response pathway in the Sono‐TT8+US group compared with the control group. (d) KEGG enrichment analysis of differentially expressed genes between the Sono‐TT8+US and control groups. (e) Immunofluorescence staining of cleaved caspase‑3, CRT, and HMGB1 in tumor sections (scale bars: 50 µm) (*n*=3). (f) qRT‑PCR analysis of Xkr8 mRNA expression in mice treated with PBS, TCT, t‑TCT, Sono‐TT8, and Sono‐TT8+US (*n*=3). (g) Western blot analysis of Xkr8 protein expression in tumor tissues after different treatments, with β‑tubulin as a loading control (*n*=3). (h) Quantitative analysis of Xkr8 protein levels corresponding to panel (g) (*n*=3). (i,j) Representative flow cytometric plots of PS externalization (Annexin V^+^) (i) and corresponding quantitative analysis (j) (*n*=3). (k–m) ELISA measurements of serum IL‑6 (k), TGF‑β1 (l), and IL‑10 (m) levels after various treatments (*n*=3). Results are presented as means ± SD. Statistical significance was calculated by one‐way ANOVA with Tukey's multiple comparisons test. (ns, not significant; *p < 0.05; **p < 0.01; ***p < 0.001; ****p < 0.0001).

To gain mechanistic insight into the antitumor effects, we performed transcriptomic profiling of tumor tissues collected from control‐ and Sono‐TT8+US‐treated mice. As illustrated by the Venn diagram, approximately 87.9% of detected genes (20,806 genes) were shared between the two groups, whereas 1,488 genes (6.2%) were uniquely expressed in the Sono‐TT8+US‐treated tumors (Figure 6a). Differential expression analysis identified 117 significantly upregulated genes and 112 significantly downregulated genes in the Sono‐TT8+US group relative to the control group (Figure 6b). To characterize the biological processes associated with these transcriptional alterations, gene set enrichment analysis (GSEA) was conducted. Notably, tumors treated with Sono‐TT8+US exhibited significant enrichment of gene signatures associated with cytokine production and inflammatory responses (Figure 6c). Moreover, Kyoto Encyclopedia of Genes and Genomes (KEGG) enrichment analysis (Figure 6d) revealed that differentially expressed genes (DEGs) were significantly enriched in multiple apoptosis‐related pathways, including positive regulation of the intrinsic apoptotic signaling pathway and activation of cysteine‐type endopeptidase activity involved in the apoptotic process. In addition, pathways associated with immune regulation, cytokine signaling, and inflammatory responses were also prominently enriched. These findings suggest that, beyond its direct cytotoxic effects, Sono‐TT8+US may exert therapeutic benefits by reshaping the tumor immune microenvironment.

Immunofluorescence staining of tumor sections showed that Sono‐TT8+US exhibited markedly elevated cleaved caspase‐3 expression compared with all other treatment groups (Figure 6e and Figure S7a–c), confirming the robust induction of apoptotic cell death. Given that apoptosis accompanied by damage‐associated molecular pattern (DAMP) release can trigger immunogenic cell death (ICD), we examined the expression of representative ICD markers. As expected, Sono‐TT8+US treatment significantly enhanced calreticulin (CRT) exposure while promoting the release of high‐mobility group box 1 (HMGB1) from tumor cells. Importantly, consistent with our in vitro findings, TPZ‐containing chemotherapy markedly increased Xkr8 expression within tumor tissues. Both qPCR and western blot analyses demonstrated that the t‐TCT group exhibited the highest Xkr8 expression, whereas Xkr8 levels were significantly reduced following treatment with Sono‐TT8+US (Figure 6f–h). To determine whether these changes were associated with PS externalization, we quantified membrane‐exposed PS by flow cytometry using Annexin V staining. As shown in Figure 6i,j, the t‐TCT group displayed the highest proportion of Annexin V‐positive tumor cells, whereas Sono‐TT8+US markedly suppressed PS externalization. Additionally, serum cytokine levels were quantified by ELISA. Compared with all other treatment groups, mice receiving Sono‐TT8+US exhibited the highest level of the pro‐inflammatory cytokine interleukin‐6 (IL‐6) (Figure 6k). By contrast, the immunosuppressive cytokines transforming growth factor‐β1 (TGF‐β1) and IL‐10 were reduced to their lowest levels following Sono‐TT8+US treatment (Figure 6l,m). These findings provide mechanistic insight into why conventional chemotherapy often elicits limited antitumor immune responses and demonstrate that blockade of the chemo‐immune checkpoint effectively shifts the TME from an immunosuppressive state toward an immunostimulatory phenotype.

**FIGURE 7:**
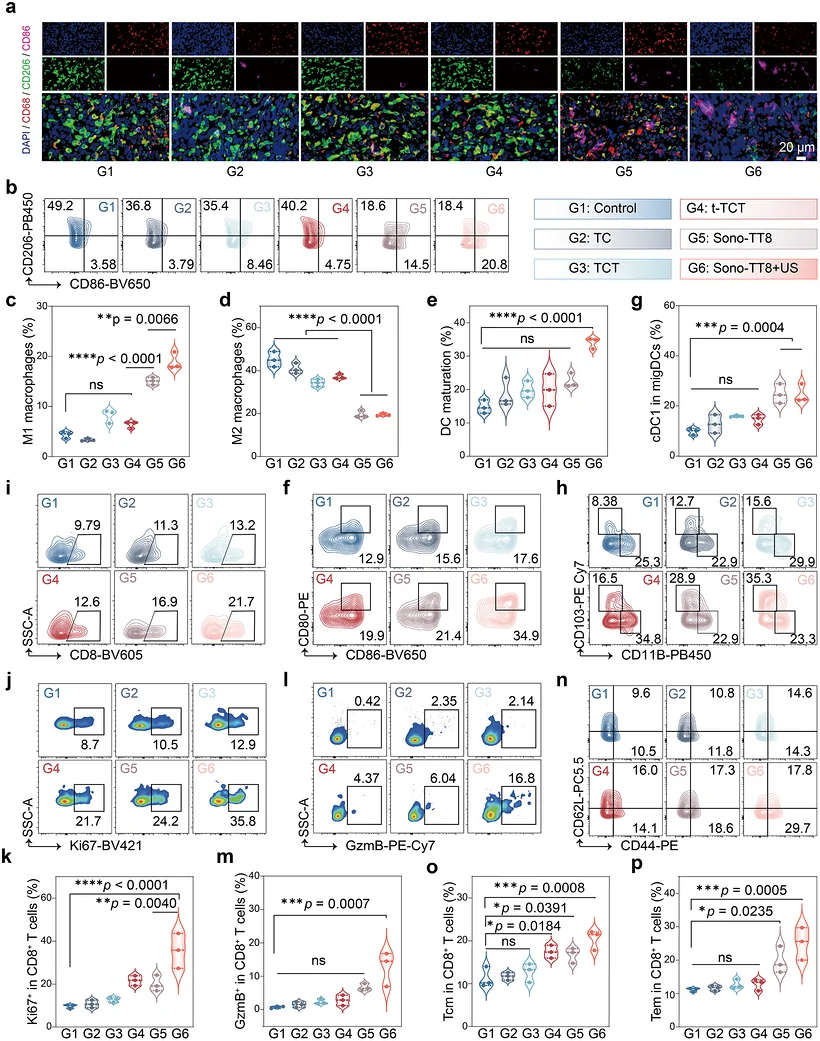
Nanoinhibitor‐mediated chemo‐immune checkpoint blockade reprogramming the tumor immune microenvironment. (a) Representative immunofluorescence images of macrophages in tumor tissues after different treatments (scale bar: 20 µm). (b) Representative flow cytometry plots of macrophages. (c,d) Quantitative analysis of M1 macrophages (CD86^+^ CD206^−^ within CD11b^+^ F4/80^+^) (c) and M2 macrophages (CD86^−^ CD206^+^ within CD11b^+^ F4/80^+^) (d) in tumors (*n*=3). (e) Quantitative analysis of mature DCs (CD80^+^ CD86^+^ within CD11c^+^ MHC‑II^+^) in TDLNs. (f) Representative flow cytometry plots of mature DCs. (g) Quantitative analysis of cDC1 (CD11b^−^ CD103^+^ within CD11c(int) MHC‑II(high)) in migratory DCs. (h) Representative flow cytometry plots of cDC1. (i) Representative flow cytometry analysis of tumor‑infiltrating CD8^+^ T cells (CD45^+^ CD3^+^ CD8^+^). (j–m) Representative flow cytometry plots of Ki67^+^ (j) and GzmB^+^ CD8^+^ T cells (l), with corresponding quantitative analyses in Ki67^+^ (Ki67^+^ within CD45^+^ CD3^+^ CD8^+^) (k) and GzmB^+^ (GzmB^+^ within CD45^+^ CD3^+^ CD8^+^) (m) CD8^+^ T cells in tumors (*n*=3). (n) Representative flow cytometry plots of memory CD8^+^ T cells in the spleen. (o,p) Quantitative analysis of central memory T cells (Tcm, CD44^+^ CD62L^+^ within CD45^+^ CD3^+^ CD8^+^) (o) and effector memory T cells (Tem, CD44^+^ CD62L^−^ within CD45^+^ CD3^+^ CD8^+^) (p) in the spleen (*n*=3). Results are presented as means ± SD. Statistical significance was calculated by one‐way ANOVA with Tukey's multiple comparisons test. (ns, not significant; *p < 0.05; **p < 0.01; ***p < 0.001; ****p < 0.0001).

Subsequently, we performed a comprehensive immune profiling of key immune populations within the TME. Immunofluorescence staining of tumor sections revealed a marked enrichment of CD206^+^ M2‐like TAMs in treatment groups lacking the siXkr8 component. Instead, tumors treated with Sono‐TT8+US exhibited prominent CD86‐associated fluorescence (Figure 7a), suggesting a shift toward a pro‐inflammatory M1‐like macrophage state. Consistent with these observations, flow cytometric analysis demonstrated that incorporation of siXkr8 reduced the proportion of CD206^+^ macrophages from over 40% to below 20%, while simultaneously increasing CD86 expression by at least 4.31‐fold relative to the control groups (Figure 7b–d). Similarly, the percentage of IFN‐γ^+^ NK cells was also significantly elevated in the Sono‐TT8+US group (29.63 ± 6.64%) compared with the control group (14.27 ± 1.01%) (Figure S8a,b). Furthermore, we performed a systematic analysis of DC populations within the tumor‐draining lymph nodes (TDLNs). The impact of PS blockade on DC maturation was initially investigated. Flow cytometric analysis revealed that treatment with Sono‐TT8+US significantly increased the proportion of mature CD80^+^ CD86^+^ DCs to 33.97 ± 1.63%, representing 2.70‐fold and 1.75‐fold increases compared with the control and t‐TCT groups, respectively (Figure 7e,f). Among DC subsets, CD103^+^ migratory DCs (cDC1s) are specialized in tumor antigen capture, cross‐presentation, and the priming of cytotoxic CD8^+^ T‐cell responses. Considering their pivotal role in orchestrating antitumor immunity, we quantified the abundance of cDC1s in TDLNs. Notably, only the Sono‐TT8 and Sono‐TT8+US groups elicited a substantial expansion of the cDC1 population, whereas cDC1 frequencies remained low in all other treatment groups (Figure 7g,h).

The increased abundance of mature DCs and cDC1s prompted us to investigate whether these innate immune changes could be translated into enhanced T‐cell‐mediated antitumor immunity [38]. Remarkably, the Sono‐TT8+US group resulted in 2.21‐fold elevation in CD8^+^ T cells, compared to the control group (Figure 7i). Specifically, Sono‐TT8+US also improved the subpopulations of CD8^+^ T cells with activation markers of Ki67^+^ (Figure 7j,k), which are defined as immune proliferation signals. In parallel, a substantial increase in granzyme B (GzmB) secreted by CD8^+^ T cells was observed in the Sono‐TT8+US group, with a frequency of 12.74 ± 5.16%, which was markedly higher than that observed in other groups (Figure 7l,m). We also assessed the impact of Xkr8 silence on establishing durable antitumor immune memory by analyzing central memory T (Tcm, CD44^+^CD62L^+^) cells and effector memory T (Tem, CD44^+^CD62L^−^) cells in the spleen of treated mice on day 42. The percentages of CD8^+^Tcm and CD8^+^Tem cells were significantly increased under Sono‐TT8+US treatment (Figure 7n–p), with only Tem population being elevated in the CD4^+^ T‐cell compartment (Figure S8c–e). It is, therefore, concluded that the blockade of the Xkr8‐PS axis converts chemotherapy‐induced anti‐tumor effect from an immunologically tolerogenic process into a potent driver of durable antitumor immunity.

### 2.7 Nanoinhibitor Synergizes With ICB to Suppress Tumor Metastatic Progression

**FIGURE 8:**
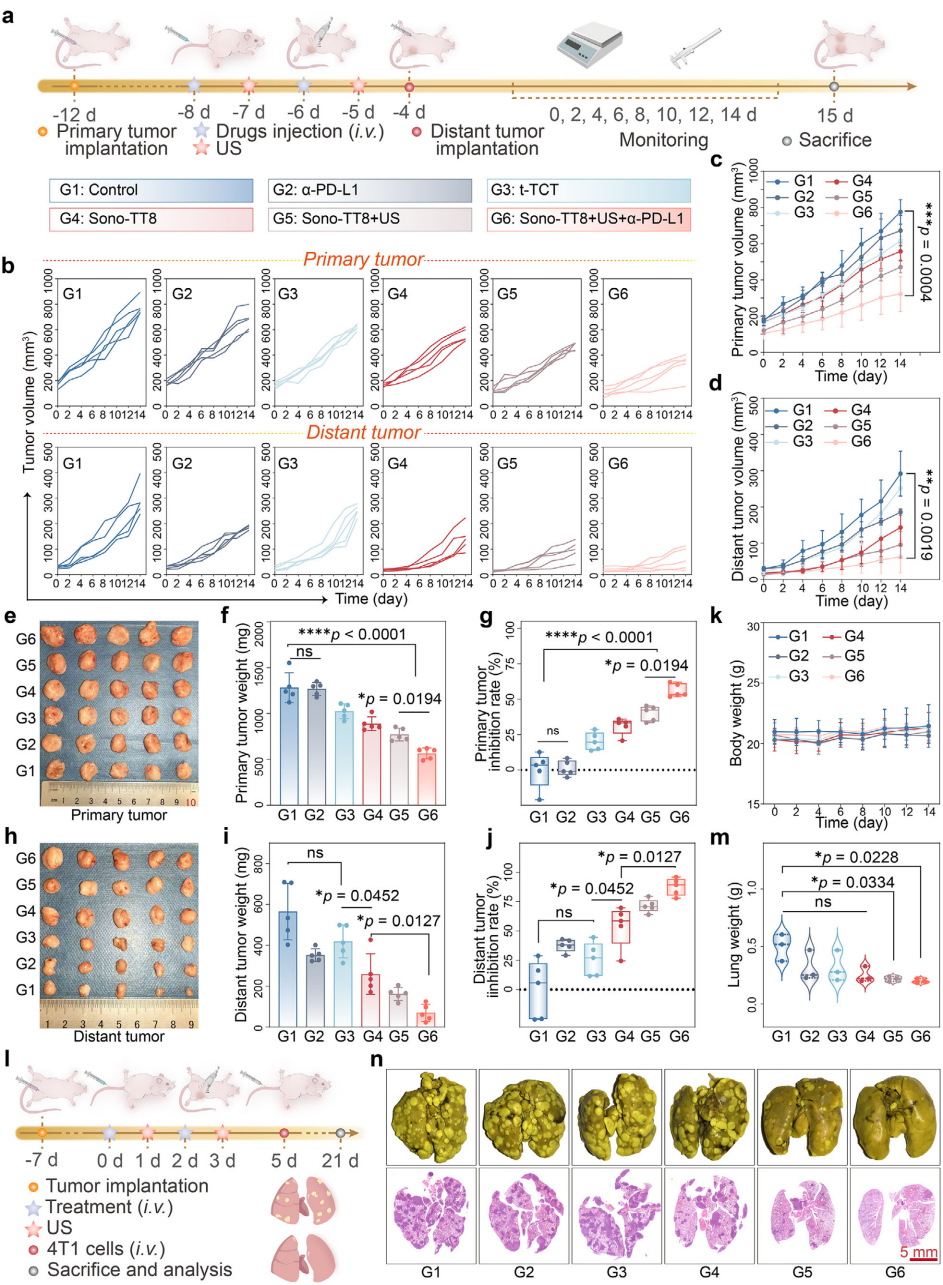
Systemic antitumor effect of nanoinhibitor combined with ICB against tumor metastasis. (a) Schematic illustration of the treatment schedule for the bilateral tumor model. (b) Individual tumor growth curves for each mouse in different treatment groups (*n*=5). (c,d) Average growth curves of primary tumors (c) and distant tumors (d) during the treatment course (*n*=5). (e–g) Representative photographs of primary tumors (e), tumor weights (f), and tumor inhibition rates (g) (*n*=5). (h–j) Representative photographs of distant tumors (h), tumor weights (i), and tumor inhibition rates (j) (*n*=5). (k) Body weight changes in bilateral tumor‑bearing mice during the 2‑week treatment period. (l) Schematic illustration of the treatment schedule for the lung metastasis model. (m) Lung weights after different treatments (*n*=5). (n) Representative photographs of Bouin's‑stained lungs and corresponding H&E staining images (scale bars: 5 mm). Results are presented as means ± SD. Statistical significance was calculated by one‐way ANOVA with Tukey's multiple comparisons test. (ns, not significant; *p < 0.05; **p < 0.01; ***p < 0.001; ****p < 0.0001).

Given that the combination of ICB and chemotherapies has been approved as a first‐line treatment for patients with metastatic breast cancer [39, 40], anti‐PD‐L1 (αPD‐L1) was selected for subsequent combination therapy studies. To evaluate the systemic antitumor effect, a bilateral 4T1 tumor‑bearing BALB/c mouse model was established, in which one tumor was designated as the primary lesion for treatment, and the contralateral tumor served as a distant, untreated tumor (Figure 8a). Although free αPD‐L1 moderately delayed the progression of distant tumors, its therapeutic effect on primary tumors was limited. In addition, Sono‐TT8+US treatment suppressed the growth of both primary and distant tumors. Notably, the addition of αPD‐L1 further enhanced the therapeutic efficacy of Sono‐TT8+US, resulting in the most pronounced inhibition of tumor growth at both tumor sites (Figure 8b–d). At the study endpoint, tumors were excised for photography (Figure 8e,h) and weighed (Figure 8f,i). Consistent with the tumor growth curves, the combination treatment exhibited the smallest tumor burden among all groups. Correspondingly, the inhibition rates of primary and distant tumors reached 56.19 ± 4.70% and 87.62 ± 7.35%, respectively, compared with the control group (Figure 8g,j), and no significant body weight loss was observed during the 14‐day treatment (Figure 8k). These results demonstrate that αPD‐L1 effectively amplifies the antitumor immune response elicited by Sono‐TT8+US, resulting in superior control of both local and distant tumors.

Encouraged by the remarkable therapeutic efficacy of the combination regimen against both primary and distant tumors, we next investigated its ability to suppress lung metastasis in 4T1 tumor‐bearing BALB/c mice (Figure 8l). On day 21 following treatment initiation, the lungs were harvested and weighed to evaluate metastatic burden. Quantitative analysis showed that treatment with free αPD‐L1, t‐TCT, or Sono‐TT8 alone failed to significantly reduce lung weight. Contrastingly, Sono‐TT8+US+αPD‐L1 markedly decreased lung weight, suggesting effective suppression of pulmonary metastasis (Figure 8m). To further assess metastatic progression, excised lungs were subjected to Bouin's fixation and histological examination. As shown in Figure 8n, numerous metastatic nodules were readily observed in the control, free αPD‐L1, and t‐TCT groups. By comparison, substantially fewer metastatic lesions were detected in mice treated with Sono‐TT8+US, while the Sono‐TT8+US+αPD‐L1 group exhibited the lowest number of pulmonary metastatic nodules and the most preserved lung architecture. Altogether, these findings demonstrate that blockade of the chemo‐immune checkpoint reprograms the immunosuppressive TME, enhances systemic antitumor immunity, and improves responsiveness to αPD‐L1 treatment, ultimately leading to effective suppression of both primary tumors and metastatic dissemination.

## 3 Discussion

Although ICB has demonstrated promising efficacy in combination with chemotherapy [41, 42], accumulating evidence suggests that activation of the caspase‐3‐Xkr8‐PS signaling axis during chemotherapy‐induced apoptosis may establish an immunosuppressive microenvironment, thereby compromising the long‐term efficacy of subsequent immune activation [10, 15, 19]. Consistent with this notion, we validated that chemotherapy‐induced Xkr8 upregulation and PS externalization drive immunosuppression, and established Xkr8 as a therapeutic target for overcoming chemo‐immune resistance. To this end, we developed an ultrasound‐responsive nanoplatform (Sono‐TT8) co‐delivering TPZ and siXkr8, which restores antigen‐presenting cell function and T‐cell activation, ultimately reversing immune resistance within the TME.

Previous studies have largely focused on neutralizing extracellular PS using annexin‐based proteins or PS‐targeting antibodies [17, 43]. Although these strategies have shown encouraging antitumor activity in preclinical studies, their clinical translation has remained limited. One possible explanation is that PS externalization is a highly dynamic process during chemotherapy‐induced apoptosis, making the timing, duration, and extent of PS exposure difficult to precisely control. Consequently, neutralizing only the extracellular PS signal may be insufficient to completely overcome the continuously regenerated immunosuppressive microenvironment. In this study, we establish Xkr8 as the primary therapeutic target and integrate chemotherapy, sonodynamic therapy (SDT), and immunotherapy into a unified nanoplatform. By silencing Xkr8 before extensive PS externalization occurs, thereby intervening at the upstream regulatory node, this design not only reduces immunosuppressive signaling but also preserves chemotherapy‐induced apoptotic killing, ultimately uncoupling tumor cell death from immune tolerance.

Conceptually, CICN shifts the paradigm from neutralizing exposed PS to preventing its externalization at the source. Interestingly, blockade of Xkr8 produced coordinated remodeling of both innate and adaptive immunity. Beyond reversing macrophage polarization, Xkr8 silencing markedly expanded migratory cDC1 populations and enhanced antigen cross‐presentation, ultimately leading to robust CD8^+^ T‐cell activation and durable immune memory. These observations support the concept that PS externalization‐induced immune suppression is not restricted to a single immune compartment but instead propagates through multiple interconnected cellular networks [44, 45]. Importantly, the Xkr8‐PS axis may also provide a complementary therapeutic opportunity for combination with canonical immune checkpoint blockade, particularly the PD‐1/PD‐L1 pathway. Whereas PD‐1/PD‐L1 blockade primarily reinvigorates pre‐existing antitumor T‐cell responses, inhibition of Xkr8‐mediated PS externalization acts at an upstream stage of the cancer‐immunity cycle. Thus, targeting these two immunosuppressive axes may exert complementary effects across distinct stages of antitumor immunity: Xkr8 silencing promotes the generation and recruitment of tumor‐reactive T cells, while PD‐1/PD‐L1 blockade preserves their effector function within the tumor microenvironment. Such mechanistic complementarity provides a compelling rationale for integrating Xkr8‐PS inhibition with PD‐1/PD‐L1 blockade to establish a more complete immune response and potentially overcome resistance to conventional immune checkpoint therapy. More broadly, Xkr8 activation represents a common downstream consequence of caspase‐dependent apoptosis rather than a TPZ‐specific event. Therefore, the concept of chemo‐immune checkpoint blockade may be applicable to a broader range of apoptosis‐inducing therapeutic modalities, including anthracycline‐ and platinum‐based chemotherapy, radiotherapy, and potentially other treatment strategies that activate caspase‐dependent apoptotic pathways. These findings suggest that targeting Xkr8 blockade may provide a broadly applicable strategy for improving combination immunotherapy across multiple cancer treatment paradigms.

## 4 Conclusion

In summary, this study demonstrates that TPZ treatment in breast cancer leads to Xkr8 upregulation and subsequent PS externalization, which drives immune resistance by impairing antigen‐presenting cell function and T‐cell activation. To overcome this chemotherapy‐induced immunosuppression, we developed an ultrasound‐responsive chemo‐immune checkpoint nanoinhibitor (Sono‐TT8) that synergistically integrates ultrasound‐triggered chemotherapy with Xkr8 silencing. Mechanistically, Sono‐TT8 simultaneously induces potent tumor cell apoptosis while preventing Xkr8‐dependent PS externalization, thereby converting chemotherapy from an immunologically tolerogenic process into an immunostimulatory one. By integrating chemotherapy with chemo‐immune checkpoint blockade, our work establishes a nanomedicine‐enabled therapeutic strategy that restores antitumor immunity, enhances responsiveness to ICB, and provides a conceptual framework for improving combination cancer immunotherapy.

## 5 Limitations

Despite the encouraging therapeutic efficacy demonstrated in this study, several limitations should be acknowledged. First, although we identified chemotherapy‐induced Xkr8 activation as a key driver of PS‐mediated immunosuppression, the upstream regulatory mechanisms responsible for chemotherapy‐associated Xkr8 transcriptional upregulation remain incompletely understood. Second, our therapeutic strategy was primarily validated in murine 4T1 breast cancer models. Given the substantial heterogeneity among breast cancer subtypes and the diverse immune landscapes across different solid tumors, further evaluation in patient‐derived xenografts, genetically engineered mouse models, and humanized immune models will be necessary to establish the broader translational applicability of chemo‐immune checkpoint blockade. Third, although Sono‐TT8 effectively enhanced the efficacy of anti‐PD‐L1 therapy, we did not directly compare this strategy with currently available PS‐targeting agents or other emerging immunomodulatory nanotherapeutics. Such comparative studies would help define the relative advantages and clinical positioning of Xkr8 inhibition. Finally, while our biosafety assessment demonstrated favorable short‐term tolerability, comprehensive long‐term pharmacokinetic, biodistribution, immunogenicity, and toxicological evaluations remain necessary before clinical translation. Addressing these limitations will further clarify the therapeutic potential of targeting the Xkr8 blockade as a broadly applicable strategy for overcoming chemotherapy‐associated immune resistance.

## 6 Experimental Section

### 6.1 Public Datasets Acquisition and Bioinformatics Analysis

In this study, mRNA‑seq data from breast cancer patients were obtained from the publicly available Zenodo (DOI: 10.5281/zenodo.15799565) and Gene Expression Omnibus (GEO, accession number GSE169246; https://www.ncbi.nlm.nih.gov/geo/) databases, in accordance with their respective access policies and publication guidelines. Data processing and analysis were performed with assistance from Scalpel Bioinformatics Analysis Studio (Nanjing, China). In addition, mRNA‑seq data from in vivo tumor samples under different treatment conditions were generated and analyzed by OE Biotech Company (Shanghai, China).

### 6.2 Materials and Reagents

Titanium tetrafluoride (TiF_4_, ≥98%), polyethyleneimine (PEI, ≥98%, *M*w = 1200), doxorubicin hydrochloride (Dox, ≥98%), and 1,3‐Diphenylisobenzofuran (DPBF, ≥97%) were purchased from Shanghai Aladdin Biochemical Technology Co., Ltd. (China). Dipalmitoyl phosphatidylcholine (DPPC) and cholesterol were purchased from Avanti Research (USA). Distearyl phosphatidylethanolamine (DSPE‐PEG2000) was obtained from Ruixi Co. (Xian, China). tLyP‐1‐DSPE‐PEG2000 was synthesized via a maleimide‐thiol coupling reaction by QYAOBIO (China Peptides Co., Ltd.) (China, Shanghai). Tirapazamine (TPZ, Catalog No. GC11150) was obtained from GlpBio (Ontario, CA, USA). 1,1’‐Dioctadecyl‐3,3,3’,3’‐tetramethylindocarbocyanine perchlorate (DiI), DEPC‑treated water, Calcein acetoxymethyl ester/propidium iodide kit (calcein AM/PI kit), reactive oxygen species assay kit (ROS assay kit), Caspase‑3 activity assay kit, ATP assay kit, NAD^+^/NADH assay kit (with WST‑8), and mitochondrial membrane potential assay kit (with JC‑1) were purchased from Beyotime Biotechnology Co., Ltd. (China). Polyvinylpyrrolidone (PVP, K29‐32, *M*w = 58,000) and copper (II) acetate monohydrate (≥99%) were supplied by Shanghai Macklin Biochemical Co., Ltd. (China). Cell Counting Kit‐8 was supplied by GlpBio (U.S.). InVivoMAb anti‐mouse PD‐L1 was acquired from Bio X Cell (U.S.). DiR was bought from AAT Bioquest (U.S.). Mouse TGF‐β1 ELISA kit, mouse IL‐10 ELISA kit, and mouse IL‐6 ELISA kit were ordered from Quanzhou Jiubang Biotechnology Co., Ltd. Fluorescein isothiocyanate (FITC)‐Annexin V was obtained from Elabscience, China, while the other antibodies used for flow cytometry in vivo were ordered from BioLegend and BD Biosciences. Cyto‐Fast Fix/Perm Buffer Set was purchased from Biolegend (San Diego, CA, USA). Murine Xkr8 siRNA (siXkr8) and FITC‐labeled siXkr8 were designed and synthesized by Tsingke Biotechnology Co., Ltd. (Beijing, China). Primers for RT‐PCR amplification of Xkr8, GAPDH, and 18S rRNA were also provided by Tsingke Biotechnology. The sequences of siXkr8 and all primers are listed in Supplementary Tables S1 and S2. For Western blot analysis, rabbit anti‐mouse Xkr8 primary antibody was obtained from Abiowell Biotechnology Co., Ltd. (Changsha, China), and mouse anti‐β‐tubulin antibody (loading control) was purchased from Boster Biological Technology Co., Ltd. (Wuhan, China).

### 6.3 Synthesis of Sono‐TT8 NPs

#### 6.3.1 Preparation of Mesoporous TiO_2_ NPs

Mesoporous TiO_2_ nanoparticles (NPs) were prepared via a hydrothermal process. In a typical synthesis, ethanol (27.6 mL), polyvinylpyrrolidone (PVP) aqueous solution (4 mL, 9.75 mg mL^−1^), HCl (250 µL, 0.1 M), and TiF_4_ (2.5 mL, 40 mM) were combined and stirred at room temperature for 90 min. The resulting mixture was then transferred to a 100 mL stainless steel autoclave and maintained at 180 °C for 3 h. After cooling, the product was collected by centrifugation and washed three times with water and ethanol. Finally, the obtained TiO_2_ NPs were freeze‐dried in a lyophilizer for 24 h before use.

#### 6.3.2 Preparation of TC NPs

TC (TiO_2_@Cu) NPs were prepared via a conventional impregnation method. Typically, 0.023 g of Cu(CH_3_COO)_2_·H_2_O powder was dissolved in 37.5 mL of water, followed by the addition of 0.25 g of TiO_2_. The suspension was stirred for 6 h. Subsequently, 3.75 mL of NaBH_4_ solution (0.062 g NaBH_4_ in 3.75 mL H_2_O) was introduced into the mixture under continuous stirring for another 4 h. The resulting Cu‑loaded TiO_2_ product was collected by centrifugation, washed several times with water, and then lyophilized for 24 h. The final nanoparticles were denoted as TC.

#### 6.3.3 Preparation of Lipid Films

Cholesterol (8 mg), DPPC (24 mg), and tLyP‐1‐DSPE‐PEG2000 (8 mg) were dissolved in chloroform. The solvent was then evaporated under reduced pressure at 45 °C for 1 h at a rotation speed of 80 rpm to yield a thin lipid film. Subsequently, the film was hydrated with 40 mL of PBS for further use. For the preparation of lipid films without the targeting peptide, the same procedure was used except that tLyP‐1‐DSPE‐PEG2000 (tLyP‐1: CGNKRTR) was replaced with DSPE‐PEG2000.

#### 6.3.4 Synthesis of Sono‐TT8 NPs

First, siXkr8 (250 µL, 20 µM) and PEI (1.2 kDa, 25.85 µL, 1 mg mL^−1^) were combined at an N/P ratio of 0.5, gently mixed, and incubated for 30 min at room temperature to allow electrostatic adsorption, forming the PEI‑siXkr8 complexes. Subsequently, the PEI‑siXkr8 complexes, TPZ solution (1.6 mL, 50 mM), and TC powder (40 mg) were mixed in DEPC‑treated water (38.6 mL) and stirred at 4 °C for 12 h. The mixture was then centrifuged to remove free PEI‑siXkr8 complexes and unloaded TPZ that were not incorporated into TC. The resulting pellet was further mixed with the pre‑prepared lipid films (40 mL) and stirred for another 12 h to achieve pore sealing. Finally, Sono‐TT8 NPs were collected by centrifugation and washed twice with DEPC‑treated water. For the preparation of control formulations, the following modifications were made: (i) the non‑targeted control of Sono‑TT8 (without tLyP‑1) was prepared by omitting tLyP‑1; (ii) t‑TCT (without siXkr8) was prepared by omitting the PEI‑siXkr8 complexes; and (iii) TCT (without both siXkr8 and tLyP‑1) was prepared by omitting both the PEI‑siXkr8 complexes and tLyP‑1.

### 6.4 Characterization

#### 6.4.1 General Characterization of Nanoparticles

The morphology, lattice fringes, electron diffraction patterns, and elemental mapping of the nanoparticles were examined using a field‐emission high‐resolution transmission electron microscope (HRTEM; Tecnai G2 F30 S‐TWIN, FEI, USA). Hydrodynamic size and zeta potential were measured by a Malvern Zetasizer Nano ZS90 analyzer based on dynamic light scattering (DLS). Brunauer–Emmett–Teller (BET) surface area was determined with a gas sorption analyzer (NOVAtouch, Quantachrome Instruments, USA). UV‑vis‑NIR absorption spectra were recorded on a UV‑vis‑NIR spectrophotometer (UV‑3600, Shimadzu, Japan). The crystalline phase was analyzed by X‑ray diffraction (XRD) using a Bruker D8 Advance diffractometer (Germany). Elemental composition was characterized by X‑ray photoelectron spectroscopy (XPS) on an ESCALAB 250Xi spectrometer (Thermo Fisher Scientific, USA). Electron spin resonance (ESR) spectra were acquired with a Bruker EMX Nano spectrometer (USA). UV‑Vis diffuse reflectance spectroscopy (DRS) was performed on a UV‑vis‑NIR spectrophotometer (UV‑3600, Shimadzu, Japan).

#### 6.4.2 PEI‐siXkr8 Complexation Assay (Agarose Gel Electrophoresis)

The complexation ability of PEI with siXkr8 was evaluated by agarose gel electrophoresis. PEI was dissolved in DEPC‑treated water at a concentration of 1 mg mL^−1^. siXkr8 (20 µM) was mixed with PEI at various N/P ratios ranging from 0 to 0.5 and incubated for 30 min at room temperature to allow electrostatic interaction. After complexation, the samples were mixed with 6× RNA loading buffer (sample: buffer = 5:1, v/v) and then electrophoresed on a 1% agarose gel at 100 V for 20 min. The gel was imaged, and the bands were analyzed.

#### 6.4.3 Drug Loading Capacity and Encapsulation Efficiency

To determine the drug loading capacity, different concentrations of TPZ solution (1, 2, 4, 6, and 8 mM) were mixed with equal amounts of TC powder (1 mg) and stirred in the dark for 12 h. After centrifugation (10,000 rpm, 15 min), equal volumes of the supernatants were collected, and their absorbance at 460 nm (the characteristic peak of TPZ) was measured using a UV‑Vis spectrophotometer. The TPZ concentration in the supernatant was calculated based on a standard calibration curve. The drug loading content (DLC) was calculated according to the following formulas:

$$\begin{array}{cc} \mathrm{DLC}\% & =(\mathrm{mass}\;\mathrm{of}\;\mathrm{drug}\;\mathrm{in}\;\mathrm{nanoparticles}/\\ & \;\mathrm{mass}\;\mathrm{of}\;\mathrm{drug}-\mathrm{loaded}\;\mathrm{nanoparticles})\times 100\% \end{array}$$

#### 6.4.4 In Vitro Drug Release Study

The release of TPZ from Sono‐TT8 NPs, with or without ultrasound irradiation (1.0 MHz, 1.3 W cm^−2^, 50% duty cycle, 5 min), was evaluated using a dialysis method. Briefly, 1 mL of the Sono‐TT8 NP suspension (containing 6.4 µmol of TPZ) with or without prior ultrasound treatment was placed into a dialysis bag (MWCO 3.5 kDa, Solarbio). The dialysis bag was immersed in 10 mL of PBS at pH 6.5 or 7.4 and incubated at 37 °C with shaking at 100 rpm. At predetermined time points (0, 0.5, 1, 2, 4, 8, 12, 24, 36, 48, 60, and 72 h), 1 mL of the external PBS was withdrawn, and an equal volume of fresh PBS was added to maintain a constant volume. The TPZ concentration in the withdrawn samples was determined by measuring the absorbance at 460 nm (characteristic peak of TPZ) using a UV‑Vis spectrophotometer, and the cumulative release was calculated accordingly.

#### 6.4.5 Detection of Singlet Oxygen

The singlet oxygen (^1^O_2_) generation capability of Sono‐TT8 NPs after drug/siRNA loading and lipid coating was assessed using the DPBF probe. Briefly, 2 mL of an aqueous solution containing Sono‐TT8 (equivalent TiO_2_ content: 100 µg mL^−1^) and DPBF (1 mg mL^−1^) was exposed to ultrasound irradiation (1.0 MHz, 1.3 W cm^−2^, 50% duty cycle) for 0, 2, 4, 6, 8, or 10 min. Subsequently, the absorption spectra from 350 to 500 nm were recorded using a multi‑mode microplate reader (M3, SpectraMax, China).

### 6.5 Cell Culture

The murine breast carcinoma cell line 4T1 cells were purchased from Boster Biological Technology, Wuhan, China (Catalog No. CX0126). HUVECs were purchased from Pricella (Wuhan, China). 4T1 cells were cultured in RPMI‐1640 supplemented with 10% fetal bovine serum (FBS) and 1% penicillin‐streptomycin. HUVECs were cultured in DMEM containing 10% fetal bovine serum (FBS) and 1% penicillin‐streptomycin. Both cell lines were maintained in a 5% CO_2_ incubator at 37°C. Subculturing was performed every 24–48 h when cells reached 80%–90% confluence.

### 6.6 In Vitro Cell Uptake Efficiency

To investigate the role of the tLyP‐1 peptide in nanoparticle‑mediated targeted delivery, Sono‐TT8 and its non‑targeted counterpart (Sono‐TT8 without tLyP‐1) were prepared using FITC (green)‑labeled siXkr8 and DiI (red)‑labeled Lipid films. For confocal microscopy, 4T1 cells were seeded in confocal dishes at a density of 1 × 10^5^ cells per dish and incubated for 24 h. The cells were then treated with DiI‑labeled nanoparticles (20 µg mL^−1^) for 1, 2, 4, and 6 h. After washing three times with PBS to remove unbound nanoparticles, the cells were fixed with 4% paraformaldehyde, and the nuclei were stained with DAPI. Fluorescence images were captured using a confocal laser scanning microscope (Nikon A1, Japan) to assess the colocalization of DiI (red) and FITC (green) signals, confirming successful siRNA encapsulation and co‑delivery. For flow cytometry analysis, 4T1 cells were seeded in 12‑well plates at a density of 2 × 10^5^ cells per well and incubated for 24 h. Cells were then treated with the same nanoparticles (20 µg mL^−1^) for 0, 1, 2, 4, and 6 h, followed by washing, collection, and resuspension in PBS. The cellular fluorescence intensity (DiI signal) was measured using a flow cytometer (Beckman CytoFLEX, Beckman Coulter, Suzhou, China) to quantify cellular uptake.

### 6.7 In Vitro Cytotoxicity Assessment

#### 6.7.1 Cell Viability Assay

4T1 and HUVEC cells were seeded in 96‑well plates and incubated with Sono‐TT8 at concentrations ranging from 0 to 40 µg mL^−1^ (quantified as TC) for 12 h to allow sufficient nanoparticle uptake. The medium was then discarded, and the ultrasound group was exposed to irradiation (1.0 MHz, 1.3 W cm^−2^, 50% duty cycle) for 2 min. Fresh nanoparticle‑free medium was added to all wells, and the cells were further incubated for 12 h. After washing three times with PBS, 100 µL of fresh medium containing 10% CCK‑8 was added to each well. Following 1 h of incubation, the absorbance at 450 nm was measured using a microplate reader.

#### 6.7.2 Live/Dead Cell Staining

4T1 cells were seeded in confocal dishes at a density of 1 × 10^5^ cells per dish and cultured for 24 h. The cells were then divided into six groups: G1: Control (PBS), G2: TC, G3: TCT, G4: t‐TCT, G5: Sono‐TT8, and G6: Sono‐TT8 + US. The cells were treated with the respective agents (equivalent TC content: 20 µg mL^−1^) for 12 h. The G6 group was additionally exposed to US irradiation (1.0 MHz, 1.3 W cm^−2^, 50% duty cycle) for 2 min. After further incubation for 12 h, the cells were co‑incubated with calcein‐AM and PI for 30 min and then observed under a CLSM.

#### 6.7.3 Intracellular ROS Detection

An ROS detection kit (2′,7′‐dichlorofluorescin diacetate, DCFH‐DA) was employed to monitor intracellular oxidative stress. Briefly, 4T1 cells were seeded in 12‐well plates at a density of 2 × 10^5^ cells per well and cultured at 37 °C for 12 h. The cells were then treated with different NPs (G1: Control (PBS), G2: TC, G3: TCT, G4: t‐TCT, G5: Sono‐TT8, and G6: Sono‐TT8+US) at an equivalent TC content of 20 µg mL^−1^ for 12 h. Group 6 was additionally exposed to ultrasound irradiation (1.0 MHz, 1.3 W cm^−2^, 50% duty cycle) for 2 min. After further incubation for 4 h, the cells were stained with DCFH‑DA (10 µM) for 20 min, and ROS levels were analyzed by flow cytometry.

#### 6.7.4 Mitochondrial Function Detection

The Sono‐TT8‑mediated mitochondrial dysfunction was evaluated by multiple assays. For confocal microscopy, 4T1 cells were seeded in confocal dishes at a density of 1 × 10^5^ cells per dish and incubated for 24 h. The cells were then divided into six groups: G1: Control (PBS), G2: TC, G3: TCT, G4: t‐TCT, G5: Sono‐TT8, and G6: Sono‐TT8 + US. The cells were treated with the respective agents (equivalent TC content: 20 µg mL^−1^) for 12 h. Group 6 was additionally exposed to ultrasound irradiation (1.0 MHz, 1.3 W cm^−2^, 50% duty cycle) for 2 min. After further incubation for 4 h, the cells were stained with JC‑1, and the intracellular distribution of JC‑1 was observed under a CLSM. For flow cytometry analysis, 4T1 cells were seeded in 12‑well plates at a density of 2 × 10^5^ cells per well and incubated for 24 h. Then, the cells were subjected to the same treatments described above, and the mitochondrial membrane potential (Δψm) was analyzed by flow cytometry. In addition, intracellular ATP and NAD^+^/NADH levels were measured using commercial ATP and NAD^+^/NADH assay kits, respectively, following the manufacturers’ instructions. The absorbance was recorded using a microplate reader, and the concentrations were calculated accordingly. The cell grouping and nanoparticle treatments were the same as described above.

#### 6.7.5 Caspase‑3 Activity Assay

4T1 cells were treated with the same agents and conditions as described above (six groups, 12 h incubation, with ultrasound irradiation for group 6). After treatment, cells were harvested, and intracellular caspase‑3 activity was measured using a commercial caspase‑3 activity assay kit (Beyotime Biotechnology, China) according to the manufacturer's instructions. The absorbance was recorded using a microplate reader, and the relative caspase‑3 activity was calculated accordingly.

#### 6.7.6 Scratch Wound Healing Assay

4T1 cells were seeded in 12‑well plates at a density of 5 × 10^5^ cells per well and cultured until reaching approximately 90% confluence. The cells were then divided into six groups: G1: Control (PBS), G2: TC, G3: TCT, G4: t‐TCT, G5: Sono‐TT8, and G6: Sono‐TT8 + US. The cells were treated with the respective agents for 12 h, and group 6 was additionally exposed to ultrasound irradiation (1.0 MHz, 1.3 W cm^−2^, 50% duty cycle) for 2 min. A sterile 200 µL pipette tip was used to create a straight scratch across the cell monolayer. The wells were washed three times with PBS to remove detached cells, and then incubated in serum‑free RPMI‑1640 medium. Images were captured under a light microscope at 0, 12, 24, and 48 h after scratching. The wound closure percentage was quantified using ImageJ software.

Migration and invasion assays: 4T1 cells were first treated with the same agents and conditions as described above (six groups, 12 h incubation, ultrasound for group 6). After treatment, cells were harvested and resuspended in serum‑free medium. For the migration assay, 2 × 10^4^ cells in 200 µL of serum‑free medium were added to the upper chamber of Transwell inserts (8‑µm pore size, Corning, USA). For the invasion assay, the upper chamber was pre‑coated with Matrigel (BD Biosciences, USA). The lower chamber contained 600 µL of complete medium with 10% FBS as a chemoattractant. After 24 h of incubation, non‑migrated or non‑invaded cells on the upper side of the membrane were removed with a cotton swab. Cells that migrated or invaded to the lower side were fixed with 4% paraformaldehyde, stained with 0.1% crystal violet, and imaged under a light microscope. The number of cells was counted in three random fields per well.

### 6.8 Silencing Efficiency of siXkr8

The siRNA targeting Xkr8 was synthesized by Tsingke Biotechnology Co., Ltd. (China), and the sequence is listed in Table S1. 4T1 cells were seeded in 12‑well plates at a density of 2 × 10^5^ cells per well and incubated overnight. The cells were then treated with the same agents and conditions as described above (six groups, 12 h incubation, with ultrasound irradiation for group 6). After treatment, the cells were further incubated in serum‑free RPMI‑1640 medium for 24 h.

For mRNA analysis, total RNA was extracted, reverse transcribed, and subjected to quantitative real‑time PCR (qRT‑PCR) using commercial kits (Hunan Accurex Biotechnology Co., Ltd., China) according to the manufacturers’ instructions. The qRT‑PCR was performed on a real‑time PCR detection system (Bio‑Rad CFX Connect, USA). The primers for Xkr8 mRNA are listed in Table S2, and the relative expression levels were calculated using the 2^−ΔΔCt^ method.

For protein analysis, cells were lysed, and protein concentrations were determined using a BCA assay. Equivalent amounts of protein were separated by SDS‑PAGE, transferred to PVDF membranes, and probed with primary antibodies against Xkr8 and β‐tubulin (loading control). After incubation with HRP‑conjugated secondary antibodies, the signals were detected using an enhanced chemiluminescence (ECL) kit and imaged with a ChemiDoc imaging system (Bio‑Rad, USA). Band intensities were quantified using ImageJ software.

### 6.9 Flow Cytometric Analysis of PS Externalization, BMDMs Polarization, and BMDCs Maturation

4T1 cells were treated with the same agents and conditions as described above (six groups, 12 h incubation, with ultrasound irradiation for group 6). After treatment, cells were either directly harvested for PS externalization analysis or co‐cultured with bone marrow‐derived macrophages (BMDMs) or dendritic cells (BMDCs) as described below. The gating strategies are shown in FigureS9a (PS externalization), FigureS9b (macrophage polarization), and FigureS9c (DC maturation).

PS Externalization: Treated 4T1 cells were harvested, washed, and stained with Annexin V‐FITC (Elabscience, China) following the manufacturer's protocol. The percentage of Annexin V‐positive cells (indicative of PS externalization) was analyzed by flow cytometry.

BMDMs Polarization: Treated 4T1 cells were co‐cultured with BMDMs in Transwell chambers (0.4‐µm pore size) for 24 h. BMDMs were then collected, blocked with anti‐CD16/32 antibody to reduce nonspecific binding, and stained with a live/dead viability dye. Surface staining was performed with fluorescently labeled antibodies against CD11b, CD86, and F4/80. After fixation and permeabilization, cells were further stained with anti‐CD206 antibody. The polarization profiles were analyzed by flow cytometry. M1 macrophages were defined as CD11b^+^ F4/80^+^ CD86^+^ CD206^−^, and M2 macrophages as CD11b^+^ F4/80^+^ CD86^−^ CD206^+^.

BMDCs Maturation: Treated 4T1 cells were co‐cultured with BMDCs in Transwell chambers (0.4‐µm pore size) for 24 h. BMDCs were then collected, blocked with anti‐CD16/32 antibody, stained with a live/dead viability dye, and then labeled with antibodies against CD11c, CD80, and CD86. The percentage of mature DCs was defined as CD80^+^ CD86^+^ cells within the CD11c^+^ population and analyzed by flow cytometry.

All flow cytometry data were acquired using a flow cytometer (Beckman CytoFLEX, Beckman Coulter, Suzhou, China) and analyzed with FlowJo software (BD Biosciences).

### 6.10 Animals and Tumor Models

Female BALB/c mice (6–8 weeks old) were purchased from Jiangsu Huachuang Sino Pharma Tech Co., Ltd. Animal‐related research adhered strictly to the protocol (Approval No. IACUC‐SAHCQMU‐2026‐0089) permitted by the Animal Ethics Committee of the Second Clinical College of Chongqing Medical University. The animals were maintained under specific pathogen‐free (SPF) conditions in SPF facilities.

For the primary tumor model, each BALB/c mouse received an injection of 1 × 10^6^ 4T1 cells suspended in 100 µL PBS into the right mammary fat pad. Eight days later, the same number of cells (1 × 10^6^ cells in 100 µL PBS) was subcutaneously inoculated into the left flank of the same mouse to generate a distant tumor, thereby establishing a bilateral 4T1 tumor‑bearing model. Tumor volumes were calculated using the formula: volume = 0.5 × length × width^2^. To generate a lung metastasis model, 5 × 10^5^ 4T1 cells in 200 µL PBS were slowly injected via the tail vein on day 12 after primary tumor inoculation.

### 6.11 In Vivo Biodistribution Study

For fluorescence imaging and biodistribution assessment, 4T1 tumor‑bearing mice were intravenously injected with 200 µL of DiR‑labeled Sono‐TT8 and its non‑targeted counterpart (Sono‐TT8 without tLyP‐1) via the tail vein (*n* = 3). Whole‑body fluorescence images were acquired using an in vivo imaging system (AniView100 Pro, Biolight Biotechnology Co., Ltd., Guangzhou, China) at 0, 2, 4, 8, 24, 48, 60, 72, and 96 h post‑injection, and the fluorescence intensity in the tumor region was recorded. After the final imaging, the mice were sacrificed, and the tumor tissues and major organs (heart, liver, spleen, lungs, and kidneys) were harvested for ex vivo fluorescence imaging; the corresponding fluorescence intensities were also recorded.

### 6.12 Antitumor Effect and Xkr8 Silencing in Primary Tumors

4T1 tumor‑bearing mice were randomly divided into six groups: G1: Control (PBS), G2: TC, G3: TCT, G4: t‐TCT, G5: Sono‐TT8, and G6: Sono‐TT8 + US. The day of the first injection was designated as day 0. Mice were intravenously administered the respective formulations at a dose of 20 mg kg^−1^ (based on TC content) on days 0, 4, and 8. Control groups received an equal volume of PBS (200 µL). On days 2, 6, and 10, the tumor region of the designated groups was exposed to ultrasound irradiation (1 MHz, 1.3 W cm^−2^, 50% duty cycle) for 4 min. During the 14‑day observation period, body weight and tumor volume were recorded every other day. At the end of treatment, tumor tissues were collected for the following analyses:

*Histology and Immunofluorescence*: Hematoxylin and eosin (H&E) staining, proliferating cell nuclear antigen (PCNA) staining, dihydroethidium (DHE) staining, and immunofluorescence staining of CRT, HMGB1, and cleaved caspase‑3.

*RNA Sequencing*: Tumors from the control and Sono‐TT8 + US groups were subjected to RNA‑seq analysis.

*qPCR and Western Blot*: Tumors from the control, TCT, t‐TCT, Sono‐TT8, and Sono‐TT8 + US groups were used to evaluate Xkr8 silencing efficiency at both mRNA and protein levels.

Flow Cytometry for PS Externalization: Tumor tissues from all six groups were processed into single‑cell suspensions. Cells were blocked with anti‑CD16/32 antibody, then stained with Annexin V‑FITC and anti‑CD45 antibody. The percentage of PS‑exposing (Annexin V^+^) cells within the CD45‑negative tumor cell population was analyzed by flow cytometry.

### 6.13 Immune System Activation of Sono‐TT8

To further investigate the in vivo immune activation induced by the nanoplatform, 4T1 tumor‑bearing mice were established and treated using the same regimen as described above. After the indicated treatments, tumors and draining lymph nodes were collected from different groups for subsequent immune analysis. The harvested tissues were minced into small fragments and incubated in digestion buffer containing collagenase (1 mg mL^−1^), hyaluronidase (1 mg mL^−1^), and DNase I (0.5 mg mL^−1^) at 37 °C for 30 min. The digested samples were then passed through a 40 µm cell strainer to obtain single‑cell suspensions, and the digestion was stopped by adding 5% FBS. The cell suspensions were centrifuged at 350×g for 5 min, and the pellets were treated with red blood cell lysis buffer to remove erythrocytes.

The resulting single‑cell suspensions were stained with appropriate antibodies for flow cytometric analysis of tumor‑infiltrating macrophages, NK cells, T cells, and dendritic cells (DCs) in draining lymph nodes. The antibody panels and gating strategies are provided in Figure S10a–e.

For additional immune assessment, tumor tissues were collected and subjected to immunofluorescence staining of tumor‑infiltrating macrophages. Meanwhile, blood samples were collected from the orbital vein, allowed to clot at room temperature for 2 h, and centrifuged at 3000 rpm for 10 min to obtain serum. The levels of TGF‑β1, IL‑10, and IL‑6 in the serum were measured using commercial ELISA kits.

To evaluate the long‑term survival and immune memory elicited by Sono‐TT8, tumor‑bearing mice were established and treated according to the protocol described above. Tumor dimensions were measured every two days, and tumor volume was calculated using the formula: volume = 0.5 × length × width^2^. Mice were considered dead when the tumor volume exceeded 1500 mm^3^. The survival of each mouse was recorded until day 42, and survival curves were plotted. At the end of the experiment (day 42), spleens were collected and processed into single‑cell suspensions. After red blood cell lysis, the cells were stained with appropriate antibodies and analyzed by flow cytometry to determine the percentages of central memory T cells (Tcm, CD44^+^ CD62L^+^) and effector memory T cells (Tem, CD44^+^ CD62L^−^) within the CD4^+^ and CD8^+^ T cell populations. The antibody panels and gating strategy are provided in Figure S10f.

### 6.14 Combination Therapy of Sono‐TT8+US with Anti‐PD‐L1

To evaluate the antitumor efficacy of Sono‐TT8 in other tumor models, bilateral tumor and lung metastasis models were established as described above.

*Bilateral Tumor Model*: As shown in Figure 8a,b, mice were randomly divided into six groups (*n* = 5 per group): G1: Control, G2: α‑PD‑L1 alone, G3: t‐TCT, G4: Sono‐TT8, G5: Sono‐TT8+US, and G6: Sono‐TT8+US+α‑PD‑L1. After primary tumor implantation, the respective nanoparticles or α‑PD‑L1 antibody were administered via tail vein injection on days 4 and 6. On days 5 and 7, mice in groups 5 and 6 were exposed to ultrasound irradiation (1 MHz, 1.3 W cm^−2^, 50% duty cycle) for 4 min. On day 8, the contralateral tumor was inoculated to establish the distant tumor. Starting from day 4 after distant tumor inoculation, the mice were observed for 14 days. During this period, body weight, primary tumor volume, and distant tumor volume were recorded every other day. At the end of the treatment, the tumor tissues were excised, photographed, and weighed.

*Lung Metastasis Model*: The lung metastasis model was established as described in section 1.9. As illustrated in Figure 8m, one week after primary tumor inoculation, treatment began and was designated as day 0. Mice were randomly divided into the same six groups. The respective nanoparticles or α‑PD‑L1 antibody was administered via tail vein injection on days 0 and 2. On days 1 and 3, mice in groups 5 and 6 were exposed to ultrasound irradiation (1 MHz, 1.3 W cm^−2^, 50% duty cycle) for 4 min. On day 5, 5 × 10^5^ 4T1 cells in 200 µL PBS were slowly injected via the tail vein. On day 21, the mice were sacrificed, and the lungs were harvested for H&E staining and metastatic nodule analysis.

*Biosafety Assessment of Sono‐TT8 In Vivo*: Healthy female BALB/c mice were randomly assigned to six groups (*n* = 5 per group): a control group (day 0, physiological saline injection), and groups that were intravenously injected with Sono‐TT8 (20 mg kg^−1^, based on TC content) and sacrificed on days 1, 3, 7, 14, and 28 post‑injection. At each time point, blood samples were collected for routine blood tests and blood biochemistry analysis. Meanwhile, major organs (heart, liver, spleen, lungs, and kidneys) were harvested for H&E staining to evaluate potential histopathological changes.

### 6.15 Statistical Analysis

Statistical analysis was performed using GraphPad Prism 10.1.2. All data were first assessed for normality using the Shapiro–Wilk test and for homogeneity of variances using Levene's test. For normally distributed data with equal variances, comparisons between two groups were performed using unpaired Student's t‑test; comparisons among multiple groups were analyzed using one‑way analysis of variance (ANOVA) followed by Tukey's multiple comparisons test. For data involving two independent variables (e.g., different concentrations and treatment groups), two‑way ANOVA was applied, followed by Tukey's multiple comparisons test for post‑hoc analysis. Non‑normally distributed data were analyzed using the Mann–Whitney U test for two‑group comparisons or the Kruskal–Wallis test for multiple‑group comparisons, and results are expressed as median with interquartile range (25th and 75th percentiles). Normally distributed data are presented as mean ± standard deviation (SD). Statistical significance was defined as ns, not significant; *p < 0.05; **p < 0.01; ***p < 0.001; ****p < 0.0001.

## Author Contributions

**Chier Du**: conceptualization, methodology, investigation, visualization, writing – review and editing. **Xun Guo**: conceptualization, methodology, writing – review and editing, supervision. **Min Xu**: investigation. **Shengzhe Hou**: investigation. **Hongjin An**: investigation. **Xuemei Tang**: investigation. **Peng Luo**: investigation. **Yi Lan**: investigation. **Hua Teng**: investigation. **Hao Yang**: investigation. **Zhiyi Zhou**: supervision, funding acquisition, project administration. **Jianli Ren**: supervision, funding acquisition, project administration. **Yunfan Liu**: writing – original draft, methodology, investigation, visualization. **Leilei Zhu**: investigation.

## Funding

This research was supported by the National Natural Science Foundation of China (Grant Nos. 82472006, 82472011 and 82171946) and the Chongqing Science and Technology & Health Commission Joint Research General Program (2025MSXM048), the Joint Project of Pinnacle Disciplinary Group, the Second Affiliated Hospital of Chongqing Medical University (2024205), and the Kuanren Talents Program of the Second Affiliated Hospital of Chongqing Medical University (KR2024G006), the National Postdoctoral Program for Innovative Talents of China (BX20250223) and the China Postdoctoral Science Foundation (2025M782415).

## Conflicts of Interest

The authors declare no conflicts of interest.

## Supporting information




**Supporting File**: advs77635‐sup‐0001‐SuppMat.docx.

## Data Availability

The data that support the findings of this study are available from the corresponding author upon reasonable request.
