# Supplementary material for: Ultrasound‐Activated Nanoinhibitors Blocking the Chemo‐Immune Checkpoint to Overcome Chemotherapy‐Induced Immune Resistance
Source: Adv Sci (Weinh). 2026 Sep 8:e77635. Online ahead of print. doi: 10.1002/advs.77635 (PMC13554490; doi:10.1002/advs.77635)
Supplement: Supplementary file 1 [file ADVS-9999-e77635-s001.docx]

Supporting Information

Ultrasound-Activated Nanoinhibitors Blocking the Chemo-Immune Checkpoint to Overcome Chemotherapy-Induced Immune Resistance

Yunfan Liu^1‡^, Chier Du^1‡^, Leilei Zhu^1^, Hongjin An^1^, Shengzhe Hou^1^, Yi Lan^1^, Xuemei Tang^1^, Min Xu^1^, Peng Luo^1^, Hua Teng^1^, Hao Yang^1^, Xun Guo^3,1*^, Jianli Ren^1 *^ and Zhiyi Zhou^2,1*^

^1^Department of Ultrasound, The Second Affiliated Hospital of Chongqing Medical University, Chongqing Key Laboratory of Ultrasound Molecular Imaging and Therapy, Institute of Ultrasound Imaging, Chongqing Medical University, Chongqing 400010, China

^2^Center for Obesity Prevention and Treatment, Chongqing Academy of Medical Sciences, Chongqing General Hospital, Chongqing University, Chongqing 401147, China

^3^Department of Ultrasound, the First Affiliated Hospital of Chongqing Medical University, Chongqing 400016, China

^‡^These authors contributed equally to this work

^*^Correspondence author: gx961125@163.com (X.G.), renjianli@cqmu.edu.cn (J. R.), zzy16966@163.com (Z. Z.)

**1. Supplementary Figures**

**Figure S1.** Per‑cell potential delta of immunosuppressive ligand‑receptor links between pre‑ and post‑chemotherapy tumor cells. Positive values indicate enhanced signaling in the post‑chemotherapy population.

**Figure S2. Physicochemical characterization of TiO_2_, TC, and Sono-TT8 nanoparticles.**

(a) SEM image of TC nanoparticles (scale bar: 1 μm). (b) XRD patterns of TiO_2_ and TC. (c) Loading efficiency of siXkr8 in Sono‑TT8. (d) Elemental mapping images of Sono-TT8 (scale bar: 100 nm). (e) TEM image of Sono-TT8 nanoparticles (scale bar: 50 nm). (f) Hydrodynamic size of TiO_2_, TC, and Sono-TT8 (n=3). (g) Zeta potential of TiO_2_, TC, and Sono-TT8 (n=3). (h) Hydrodynamic size of Sono-TT8 in various mediums mimicking physiological conditions (n=3). (i) Time-dependent UV-Vis absorption spectra of DPBF for ¹O₂ detection with Sono-TT8.

**Figure S3. Cellular uptake, biocompatibility, and anti-migration/invasion effects of Sono-TT8 NPs.**

(a–c) Flow cytometry histograms and MFI quantification of Dil-labeled non-targeted nanoparticles (Dil-Sono-TT8 without tLyP1) and targeted nanoparticles (Dil-Sono-TT8 with tLyP1) internalized by 4T1 cells over 6 h (n=3). (d-e) Western blot analysis of FDX1 protein expression in 4T1 cells after PBS, TC and Sono-TT8 treatments, with β‑tubulin as a loading control (n=3). (f) CCK-8 assay showing viability of 4T1 cells and HUVECs after incubation with various concentrations of TC (n=3). (g) Representative flow cytometry histograms of intracellular ROS levels measured by DCFH-DA staining in 4T1 cells. (h) Representative flow cytometry histograms of mitochondrial superoxide levels in 4T1 cells after various treatments. (i) Quantification of MitoSOX‑Red fluorescence intensity from three independent experiments (n=3). (j) Representative flow cytometry plots of JC-1 staining showing mitochondrial membrane potential (ΔΨm) in 4T1 cells. (k-m) Scratch wound-healing and Transwell migration/invasion assays of 4T1 cells treated with different formulations (n=3). Statistical significance was calculated by one-way ANOVA with Tukey’s multiple comparisons test. (ns, not significant; **p* < 0.05, ***p* < 0.01, ****p* < 0.001. *****p* < 0.0001). (G1: Control, G2: TC, G3: TCT, G4: t-TCT, G5: Sono-TT8, G6: Sono-TT8+US)

**Figure S4.** Representative brightfield microscopy images of BMDM morphology (n=3. Red arrows indicate M2‑like macrophages).

**Figure S5. In vivo biocompatibility evaluation of Sono-TT8 NPs.**

(a, b) Hematological parameters (a) and serum biochemical markers (b) measured in mice on days 0, 1, 3, 7, 14, and 28 after intravenous injection of Sono-TT8 (n=5). (c) Representative H&E-stained sections of major organs (kidney, heart, lung, liver, spleen) from treated mice at the indicated time points (n=3).

**Figure S6. In vivo circulation and tumor accumulation of DiR‑labeled Sono‑TT8.**

(a) Plasma fluorescence intensity of DiR‑labeled Sono‑TT8 at indicated time points after intravenous injection (n=3). (b) Time-dependent fluorescence intensity in the tumor region after intravenous injection of Sono-TT8 with tLyP1 (targeted) or Sono-TT8 without tLyP1 (non-targeted) nanoparticles, measured at 0, 2, 4, 8, 24, 48, 60, 72 and 96 h (n=3). (c) Ex vivo fluorescence intensity of major organs (liver, heart, spleen, lung, kidney) and tumor at 96h (n=3). Statistical significance was calculated by one-way ANOVA with Tukey’s multiple comparisons test. (ns, not significant; **p* < 0.05, ***p* < 0.01, ****p* < 0.001. *****p* < 0.0001).

**Figure S7.** Quantitative analysis of immunofluorescence staining of (a) Cleaved caspase‑3, (b) HMGB1, and (c) CRT in tumor sections (n=3). Statistical significance was determined using the Kruskal‑Wallis test followed by Dunn’s post‑hoc test. *p < 0.05. (G1: Control, G2: TC, G3: TCT, G4: t-TCT, G5: Sono-TT8, G6: Sono-TT8+US)

**Figure S8. Intratumoral NK cell activation and splenic long-term CD4^+^ T cell memory responses.**

(a) Representative flow cytometry plots of IFNγ^+^ NK cells (IFNγ^+^ within CD3^-^ CD49b^+^) in the tumor. (b) Quantification of IFNγ+ NK cell percentages among total NK cells (IFNγ^+^ within CD3^-^ CD49b^+^) (n=3). (c) Representative plots of CD4^+^ T cell memory subsets in the spleen. (d, e) Quantitative analysis of (d) central memory T cells (Tcm, CD44⁺ CD62L⁺ within CD45⁺ CD3⁺ CD4⁺) and (e) effector memory T cells (Tem, CD44⁺ CD62L⁻ within CD45⁺ CD3⁺ CD4⁺) in the spleen (n=3). Statistical significance was calculated by one-way ANOVA with Tukey’s multiple comparisons test. (ns, not significant; **p* < 0.05, ***p* < 0.01, ****p* < 0.001. *****p* < 0.0001). (G1: Control, G2: TC, G3: TCT, G4: t-TCT, G5: Sono-TT8, G6: Sono-TT8+US)

**Figure S9. In vitro gating strategies for different cell populations.** (a) Annexin V⁺ 4T1 tumor cells. (b) Bone marrow‑derived macrophages (BMDMs). (c) Bone marrow‑derived dendritic cells (BMDCs). For (b) and (c), fluorescence minus one (FMO) controls were performed to determine the cut‑off point.

**Figure S10. In vivo gating strategies for immune cell populations isolated from tumor, lymph node, and spleen of 4T1 tumor‑bearing mice.** (a) Annexin V⁺ 4T1 tumor cells in tumor tissue. (b) Tumor‑associated macrophages (TAMs). (c) Tumor‑infiltrating T cells. (d) Tumor‑infiltrating NK cells. (e) Dendritic cells (DCs) in lymph nodes. (f) Effector memory T cells (Tem, CD62L⁻ CD44⁺) and central memory T cells (Tcm, CD62L⁺ CD44⁺) among splenic CD4⁺ and CD8⁺ T cells on day 42.

**2. Supplementary Tables**

**Table S1. Sequences of siXkr8**

| Sequences (5’ to 3’) |  |
| --- | --- |
| Sense | GGUGUUUGCACGGAAUGCA(dT)(dT) |
| Anti- sense | UGCAUUCCGUGCAAACACC(dT)(dT) |

**Table S2. Sequences of Xkr8, GAPDH and 18s primers.**

| Sequences (5’ to 3’) |  |
| --- | --- |
| Xkr8-F | CAGAGACCGATGGTAATCCAGC |
| Xkr8-R | AGCTCACACTGGTGCTGGCAAT |
| GAPDH-F | CATTATCGGCCTTGACTGTG |
| GAPDH-R | TGGCAAAGTGGAGATTGTTG |
| 18s-F | GTAACCCGTTGAACCCCATT |
| 18s-R | CCATCCAATCGGTAGTAGCG |

**Table S3.** **siRNA loading content of representative nanoplatforms.**

| Year | Nanoplatforms | Main components | siRNA loading content (%) | Journal |
| --- | --- | --- | --- | --- |
| 2024 | Chol-MEs/siPLK1 | cholesterol  exosomes  siPLK1 | 4.00 | Nature Nanotechnology^[1]^ |
| 2024 | Ap-DSNPs | Poly-lactic-glycolic acid  DOX  NGF siRNA | 1.01 | Biomaterials^[2]^ |
| 2025 | MACP siTNF-α NPs | polyamidoamine polymers  Prussian blue  siTNF-α | 0.33 | Advanced Science^[3]^ |
| 2026 | LOTR NPs | Lipid nanoparticles  MGMT-siRNA  Temozolomide | 31.25 | ACS nano^[4]^ |
| 2026 | AEVs@siRNA | siSerpina3n  extracellular vesicles | 4.6 | Journal of Controlled Release^[5]^ |

**Reference:**

(1) Y. Zhuo, Z. Luo, Z. Zhu, et al., "Direct cytosolic delivery of siRNA via cell membrane fusion using cholesterol-enriched exosomes," *Nat. Nanotechnol.* **19** (2024): 1858-1868,

<https://doi.org/10.1038/s41565-024-01785-0>

(2) S. Zuo, Z. Wang, X. Jiang, et al., "Regulating tumor innervation by nanodrugs potentiates cancer immunoche motherapy and relieve chemotherapy-induced neuropathic pain," *Biomaterials* **309** (2024): 122603,

<https://doi.org/10.1016/j.biomaterials.2024.122603>

(3) H. Xie, X. Huang, B. Li, et al., "Biomimetic Nanoplatform for Targeted Rheumatoid Arthritis Therapy: Modulating Macrophage Niches Through Self-Sustaining Positive Feedback-Driven Drug Release Mechanisms," *Adv. Sci. (Weinh)* **12** (2025): e2416265,

<https://doi.org/10.1002/advs.202416265>

(4) F. Wang, Y. Li, J. Xu, et al., "DNA Repair Enzyme Regulation Strategy for Enhanced Pancreatic Neuroendocrine Tumor Therapy via Targeting siRNA-Lipid Nanoparticles," *ACS Nano* **20** (2026): 11108-11125,

<https://doi.org/10.1021/acsnano.5c21452>

(5) J. Zhang, G. Xu, J. Chen, et al., "Astrocyte-derived exosome-mediated siRNA delivery combined with quercetin-Mn complex promotes neural repair in spinal cord injury," *J. Control. Release* **396** (2026): 115104,

<https://doi.org/10.1016/j.jconrel.2026.115104>
